# Older Individuals Convicted of Sexual Offenses: A Literature Review

**DOI:** 10.1177/10790632211024244

**Published:** 2021-06-26

**Authors:** Rebecca L. Crookes, Carlo Tramontano, Sarah J. Brown, Kate Walker, Hayley Wright

**Affiliations:** 1 Coventry University, UK; 2 University of the Sunshine Coast (USC), Queensland, Australia; 3 University of the West of England (UWE), Bristol, UK; 4 Northamptonshire Healthcare NHS Foundation Trust, Northampton, UK

**Keywords:** older, aging, lifespan, sex offending

## Abstract

The population of older individuals convicted of sexual offenses (OSOs) is rapidly increasing. However, we have little understanding of their characteristics (e.g., demographic, psychological, individual, offense, and risk) and needs. To identify any similarities or differences that are unique to older individuals convicted of sexual offending, it is important to compare such characteristics across the adult lifespan. Therefore, the aim of this systematic review was to specify and synthesize the current knowledge of characteristics across the adult lifespan of the population of individuals convicted of sexual offenses. Five databases were searched and 10,680 results were screened, resulting in 100 studies included in the final review. The findings were grouped into four emergent themes: age of onset and prevalence; offender and offense characteristics; age and the risk of reoffending; and treatment. Implications of the findings from this review are discussed in relation to future research and clinical practice.

## Introduction

The relationship between age and general criminal behavior has been widely researched, with findings universally demonstrating that as age increases, the likelihood of an individual offending decreases (e.g., Barbaree et al., 2009; Farrington, 1986; Hirschi & Gottfredson, 1983). However, there has been a rapid increase in the number of older prisoners over recent decades, which contradicts the effect that aging has on offending behavior. This trend has been reported worldwide (Walmsley, 2016) including in countries such as France (PMJ5, French Prison Authority, cited in Combalbert et al., 2018), Japan (Bedard et al., 2017; Williams, Goodwin, et al., 2012), the United States (Bureau of Justice Statistics, 2016), Australia (Baidawi et al., 2011), and Canada (Penal Reform International, 2015). For instance, within the United Kingdom, the number of prisoners older than 50 years has increased proportionately more than any other age group, with the number of prisoners older than 50 years being 169% higher in December 2016 than in 2002. At the end of March 2017, the total prison population in England and Wales was approximately 85,500 (Allen & Watson, 2017). Of these, approximately 10% (8,386 prisoners) were aged between 50 and 59 years, and 5% (4,582 prisoners) were older than 60 years.

This increase can be attributed to an aging society, longer custodial sentences, (Howse, 2003), and an increase in the number of older individuals being convicted and sentenced, particularly for historical sexual offenses (House of Commons Justice Committee, 2013). Generally, the older imprisoned population can be divided into the following distinct groups: (a) individuals who received long custodial sentences when young and have aged in prison; (b) those who have repeatedly committed offenses and have repeatedly received custodial sentences throughout their lifetime (i.e., recidivist offenders); (c) individuals convicted of historical offenses, whereby the individual is convicted long after the offense was committed (i.e., historical offenders; House of Commons Justice Committee, 2013); and (d) those who committed their first crime at a later stage in life (i.e., first-time offenders; Smyer et al., 1997; Uzoaba, 1998).

Despite the increase in this older population, it can be questioned whether prisons address the needs of the older prisoners, with previous research highlighting that the needs and requirements of older prisoners differ from those of younger prisoners (Fazel, 2000; Kakoullis et al., 2010). For instance, in the United Kingdom, the HM Inspectorate of Prisons report (2004) found that the National Offender Management Service (now Her Majesty’s Prison and Probation Service [HMPPS]) and prisons provided a lack of care for older prisoners in the United Kingdom. It was reported that as a prisoner ages, more barriers to an active life, more needs for their physical and mental health, and a lower likelihood of living and functioning with dignity were apparent. These issues were reflected in the follow-up 2008 report by the HM Chief Inspector of Prisons and, more recently, in the House of Commons Justice Committee (2013) report examining older prisoners, which recommended better communication between the prison service and health care.

When compared with their younger counterparts, older prisoners display differences in individual and offense characteristics. For instance, mental health differences include higher levels of physical and psychotic comorbidities (Fazel, Hope, O’Donnell, & Jacoby, 2001); being 5 times more likely to suffer from depression (Fazel, Hope, O’Donnell, Piper, & Jacoby, 2001); a higher prevalence of dementia, affective psychosis and cerebral lesions; lower rates of personality disorders and schizophrenia (Fazel & Grann, 2002); and higher rates of affective disorders and alcohol abuse (Davoren et al., 2015; Kakoullis et al., 2010) but lower levels of illicit substance use (Kakoullis et al., 2010). When examining individual and offense characteristics, Kakoullis et al. (2010) found that older prisoners were more likely to have been married and had children, have a higher level of education, and have earned a higher income in comparison with younger prisoners. In addition, older individuals committed fewer burglary, robbery, and theft offenses, as well as drug offenses and violent offenses (Home Office, 2008).

It has been widely noted that individuals convicted of sexual offenses are overrepresented among older prisoners; an effect that has, again, been found worldwide (Fazel, Hope, O’Donnell & Jacoby, 2001; Morton, 1992; Uzoaba, 1998). Yet, despite the plethora of research that has been conducted with individuals convicted of sexual offenses, research specifically examining older individuals convicted of sexual offenses (OSOs) has remained sparse. This could have a negative impact on the interventions provided to this group, as the most effective treatment programs follow the Risk, Needs and Responsivity principles (Andrews & Bonta, 2007), but it is challenging to tailor treatment to the needs of this population when little is known about their characteristics and treatment needs. Moreover, theories, intervention programs, and risk assessment methods have been developed using the findings of research conducted primarily on individuals in their early to mid-adulthood; hence practice, or some aspects of such treatment and interventions, might not be appropriate or effective for individuals in older adulthood, particularly for those who began offending in mid to late adulthood, or have been convicted of historical offenses.

A recent systematic literature review by Chua et al. (2018) examined seven publications that provided data on first-time OSOs aged 65 years and older. The findings demonstrated that (a) the most common diagnosis among this population was dementia/neurocognitive disorder (though assessments for such disorders were not routinely delivered in the review); (b) victims of first-time OSOs tended to be vulnerable persons (e.g., children, the elderly, or individuals with an intellectual disability); and (c) there are two types: those who had repeatedly offended throughout their lifetime but were convicted at a later stage of life, and individuals with a neurocognitive disorder (i.e., those experiencing dementia could exhibit sexually disinhibited behavior). Although this review provided a good starting point for future exploration into this population, with the authors concluding that better designed studies are needed that examine the characteristics of first-time OSOs, it does not consider other subpopulations within the more broad older sexual offending population.

As such, there is evidently a gap in our knowledge with important implications for assessment and treatment. It is essential to establish what is currently known within the existing literature concerning OSOs (particularly those committing and being convicted of their first sexual offense in mid to late adulthood and those convicted of nonrecent offenses).Therefore, the aims of this systematic literature review were to (a) specify the current knowledge of characteristics (e.g., demographic and individual, psychological, offense, risk, and treatment) in individuals convicted of sexual offenses aged 60 years and older and (b) explore the similarities and differences of such characteristics among adults convicted of sexual offenses across the lifespan, to establish what we know about this population to inform practice and identify gaps in our knowledge and direct future research.

## Search Strategy

The Preferred Reporting Items for Systematic Reviews and Meta-Analyses (PRISMA) guidelines (Moher et al., 2009) were used to organize and report this systematic review. As required by the university to which the first author is affiliated, ethical approval to conduct the review was granted by the corresponding ethics review board.

### Inclusion and Exclusion Criteria

Studies included in the final analysis focused on OSOs, on age differences across the adult sexual offending population, or on comparisons among age bands groups (representing the adult lifespan). Only research/reports published after 1990 were included, as this is deemed the benchmark for the “what works” literature. The term “what works” was first used in 1974 (Martinson, 1974), but gathered momentum when researchers noted there was no clear evidence of any reliable method of reducing reoffending (House of Commons Library, 2012). In 1995, McGuire and Priestley found that punitive measures had done little to stop offending, which consequently raised the debate as to “what works” in preventing reoffending. Subsequently, there was a boost in influx of research evaluating the effectiveness of treatment programs being delivered.

The review also only included research that focused on adult male individuals convicted of sexual offenses. Despite the body of research on sexual offending, research on females convicted of sexual offenses remains relatively sparse. In addition, according to international statistics, female sexual offenses only contribute to 4% or 5% of all sexual offenses (Cortoni et al., 2010). Therefore, only studies examining males convicted of sexual offenses were included in this review.

Furthermore, research has demonstrated adolescents are a distinct population from adults, with a number of differences between the two age groups (McKillop et al., 2015). As such, only studies using samples of adults convicted of sexual offenses were included. Similarly, given individuals with learning disabilities convicted of sexual offenses have been found to have different risk assessment and treatment needs (Craig & Hutchinson, 2005; Henson, 2009), this review only includes studies exploring non-learning-disabled populations.

Papers included in the review were peer-reviewed articles, books/book chapters, and dissertations/thesis written in English. Moreover, primary studies and meta-analyses were included. Despite meta-analyses being included, systematic literature reviews were not included as to avoid the duplication of studies.

It is important to note that researchers have long debated the age at which a prisoner becomes “older” or “geriatric,” with some research defining “older prisoners” as being older than 50 years and other studies using 55 years and older (Williams, Stern, et al., 2012). It is difficult to provide a definition of older age among the population convicted of offenses, as evidence has suggested that prisoners age more rapidly compared with nonoffending populations; prisoners’ physiological age, on average, is 10 to 15 years older than their chronological age (Williams, Goodwin, et al., 2012). When specifically focusing on aging and individuals convicted of sexual offending, Marshall (2010) defined “older” as 60 years of age and older, as

it is around this age that a number of later life issues that may have an impact on a propensity to sexually offend begin to confront men, such as retirement, loss of a loved one, health difficulties, or difficulties of sexual functioning. (Marshall, 2010, p. 30)

### Search Methods

Five databases were searched: Academic Search Complete; CINAHL; PsycINFO; ProQuest Nursing and Allied Health; and PubMed. These databases were considered the most relevant for addressing the research question for this study, as they provide the best coverage for psychological (e.g., health, social, and forensic) and health-related literature available for this topic. Similarly, systematic reviews conducted within the field of forensic psychology have reported using the same databases (Brown & Kloess, 2020; Gerard et al., 2014; Schmucker & Lösel, 2008). Variants of the following search terms were generated and used to search the databases: (sex* offen*) AND (“age” OR “aging” OR “ageing” OR “elder*” OR “old*”). The search terms remained broad, as to ensure the inclusion of the wide range of studies in which individuals of different ages convicted of sex offenses have been examined. It should be noted that not only studies that had a main aim/focus on aging within the population of individuals convicted of sexual offenses/OSOs but also studies investigating sexual offending more generally were searched and reviewed; studies were reviewed if the included sample represented a wide age range (18 to older than 60 years), and findings pertaining to age were reported.

### Data Collection and Analysis

**Figure 1.**
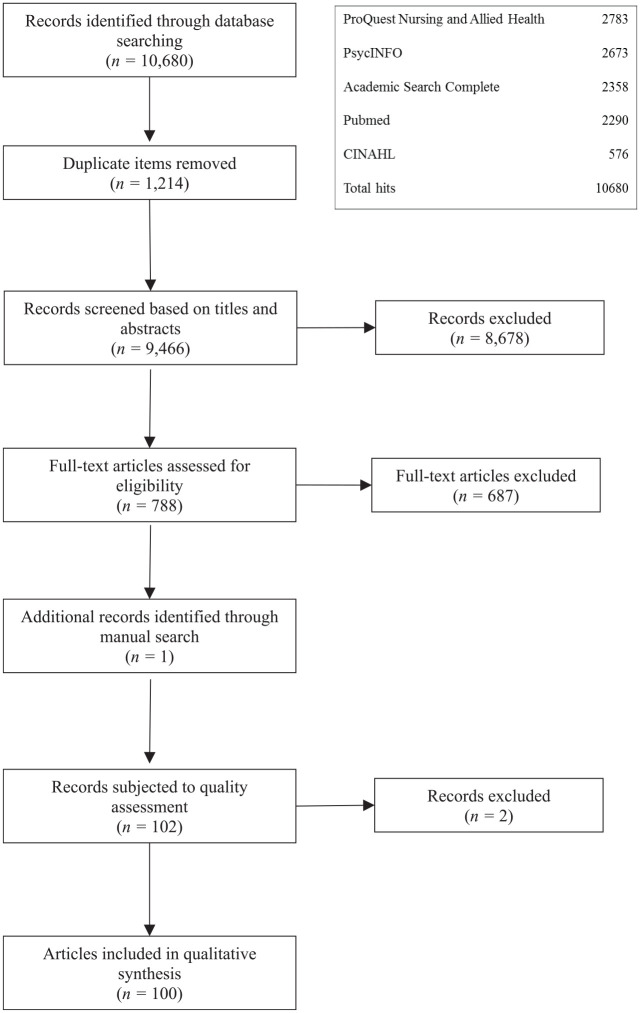
Search strategy of the systematic review.

The first search was conducted on December 11, 2017. Figure 1 outlines the implemented search strategy.

The initial search generated 10,680 results. After removing duplications (*n* = 1,214) and applying the inclusion criteria (initially excluding *n* = 9,466), there were 877 studies that were relevant based on the screening of titles and abstracts.

The first author initially screened all the studies. To ensure the reliability of the selection of papers, the third author applied the inclusion/exclusion criteria to 10% of the 10,680 hits (*n* = 1,068). Out of the number of papers reviewed, there was a disagreement on only three. Otherwise, there were no major areas of disagreements, and any minor differences were addressed through discussion and further consideration of the papers in question by both authors until an agreement was reached.

Full-text reviews were then employed on the remaining 877studies, applying the inclusion/exclusion criteria. In cases where the full texts could not be accessed or sourced, the research team submitted document supply requests and interlibrary loans through the university library, and also directly requested the studies from the authors via email or ResearchGate. However, for 22 studies, the full text could not be accessed or sourced. Moreover, out of the 81 dissertations, only 14 were freely available and obtained.

As such,8,678 studies were excluded and the inclusion criteria were applied to788 full-text papers, excluding 688 studies. Finally, a manual search of websites such as Google Scholar and ResearchGate (searching for additional published papers and gray literature), as well as of the relevant papers’ reference lists, was undertaken for papers not identified from the database search that met the inclusion criteria. Only one further article was found and included in this review, resulting in 100 papers.

### Quality Assessment

Studies were subjected to quality assessment using the quality assurance checklist “Mixed Methods Appraisal Tool” (MMAT; Hong et al., 2018). The MMAT was initially developed in 2009 (Pluye et al., 2011) and most recently revised in 2018; the latter version was used for this review. The tool was designed to assess the risk of bias, and to appraise and describe the methodological quality of qualitative, quantitative, and mixed methods studies. Given the wide range of methodologies represented in the included studies, the use of the MMAT was appropriate for this review. However, meta-analyses cannot be assessed using this tool.

The process of using the MMAT is divided into two stages: The first consists of two screening questions (“are there clear research questions” and “do the collected data allow to address the research questions?”), and the second consists of five categories (based on the design of the study, including qualitative studies, quantitative randomized control studies, quantitative nonrandomized studies, quantitative descriptive studies, and mixed methods), each including five criteria depending on the design of the study (e.g., “are the participants representative of the target population?,” “is the qualitative approach appropriate to answer the research question?” and “are the measurements appropriate?”). The assessor rates the studies by checking “yes,” “no,” or “can’t tell.” However, it is discouraged by the designers of the tool to calculate an overall quality score, and therefore, excluding studies that have a low-quality rating should be avoided (Hong et al., 2018). As such, only two papers were excluded as a result of the quality assessment, as they did not meet the requirements of the two screening questions (to finally include *n* = 100 studies in the review).Although it is discouraged to provide an overall quality score (Hong et al., 2018), the researchers deemed 42 studies as five star, 22 as four star, 19 as three star, seven as two star, and three as one star (median rate: 4.00; *M* score: 4.00, *SD* = 1.12). As six of the studies were meta-analyses and one was a book chapter, these could not be quality assessed but are still included in the review, given vital information pertinent to the topic of this review was provided. All included papers were initially assessed by the first author, and 10 papers were independently rated by the second author to assess the reliability of the evaluation. The agreement between the two raters resulted in an almost perfect agreement (Cohen’s k = .86).

A summary table of the included studies and the quality ratings is available as a Supplement (please see Supplemental Summary Table for “Older Individuals Convicted of Sexual Offenses: A Literature Review”). Please note that the presented table only includes key findings from the studies and is not exhaustive of the results reported. Where studies are discussed in the findings presented below, the MMAT rating is denoted next to the citation by the number of asterisks.

### Data Extraction and Synthesis

The final studies were reviewed and categorized into research themes. A meta-analysis was not possible, given the broad range of study aims, designs, variables, and measures used. A narrative synthesis of the extracted data was therefore undertaken, from which three areas of research focus arose: individual and offense characteristics; age and risk of reoffending; and treatment. Studies were qualitatively weighted in terms of the study design, sample size, geographical reach, and findings; a higher weight was given to meta-analyses and quantitative studies with representative samples, and lower weights were given to qualitative studies or quantitative studies with restrictive sample size and scope. As such, the largest, more robust studies are reported first and supported by smaller, less robust studies. The weighting process was undertaken collaboratively throughout by the first and second authors and completed by consensus; any disagreements were discussed until a mutual agreement was reached. The qualitative weighting was undertaken in addition to the MMAT ratings as requested by the anonymous peer reviewers of earlier versions of the manuscript, to accurately present the studies in this review.

## Findings

In total, 100 studies were included in the final review. Collectively, samples examined both those convicted of offenses against children and those convicted of offenses against adults, as well as individuals who had committed both contact (including rape and child molestation) and noncontact (e.g., involving child sexual abuse material) offenses. Studies were sourced from a wide range of countries including Canada (*n* = 38), the United States (*n* = 23), the United Kingdom (*n* = 13), Australia (*n* = 7), Austria (*n* = 4), New Zealand (*n* = 5), Sweden (*n* = 3), Italy (*n* = 2), Spain (*n* = 2), Germany (*n* = 1), Brazil (*n* = 1), and France (*n* = 1).

Of the 100 studies, 87 implemented a quantitative methodology, including using phallometric assessments, administering various psychometric instruments and self-report questionnaires (e.g., Hare’s Psychopathy Checklist, IQ tests, and risk assessment tools), and rigorous meta-analyses. Most commonly (*n* = 65), secondary data analysis was undertaken, with studies using case file data from official sources, such as police/national crime databases, prison or facility records, and other sources of case records. This information consisted of demographic information for those convicted of sexual offenses, risk assessment outcomes, and data on reoffending. Only six studies used a qualitative approach, all with semi-structured interviews, and five employed a mixed methods design, typically using interviews that were supplemented by questionnaires, secondary data (i.e., case file data), or psychometrics and other measures (e.g., measuring levels of empathy). The reference table below provides an overview of the studies included in each theme. The numbers under the “corresponding study” column refer to the numbered citation denoted in brackets at the end of the citation in the reference list.

### Characteristics of Individuals Convicted of Sexual Offenses and the Offense

#### Age of onset

Eighteen studies examined the age of onset of offending behaviors and the prevalence of OSOs. Countries included the United States (*n* = 6), Canada (*n* = 5), Australia (*n* = 4), Austria (*n* = 1), Germany (*n* = 1), Spain (*n* = 1), and the United Kingdom (*n* = 1). Only two studies were deemed “lower quality,” and 11 were deemed “higher quality” (as indicated in Table 1).

**Table 1.** Reference Table for Studies Included in Each Theme.

Theme	Number of studies	Corresponding study
Offender and offense characteristics	61	1, 5, 8, 9, 10, 11, 12, 13, 15, 16, 17, 18, 19, 20, 21, 22, 25, 26, 27, 28, 29, 30, 31, 32, 34, 35, 38, 44, 45, 48, 51, 52, 53, 54, 55, 61, 64, 65, 66, 67, 68, 69, 70, 71, 72, 76, 79, 80, 83, 84, 90, 93, 94, 95, 96, 99, 100
Aging and risk of reoffending	44	2, 3, 6, 7, 14, 23, 24, 29, 33, 36, 37, 39, 40, 41, 42, 43, 46, 47, 49, 50, 57, 58, 59, 60, 62, 63, 67, 73, 74, 75, 77, 78, 80, 81, 82, 85, 86, 87, 88, 89, 92, 94, 97, 98
Treatment	3	56, 67, 75

Generally, the age-crime curve identifies onset in early adolescence, peaking in early adulthood and then declining. However, it can be argued that this pattern is not evident in the population of individuals convicted of sexual offenses, given that seems to be a range of ages in relation to onset. Collectively, the studies suggest that adult age of onset of sexual offending ranged from mid-20s to mid-30s, demonstrating that the onset of sexual offending declines as individuals age, thus supporting the age-crime effect. For instance, Mathesius and Lussier (2014)***** reported the mean age of actual onset of sexual offending, in their sample, was 32.1 years (*SD* = 8.8) and ranged from 14.2 to 73.1 years. Indeed, Smallbone et al. (2008) suggested that there are two distinct populations based on age of onset of sexual offending: (a) individuals who first sexually offended during adolescence but stopped offending into adulthood and (b) most adult offenders who start to offend during adulthood had a peak risk period of late 30s. Moreover, in the study by Doyle et al. (2011)*** who found that in their sample of 50 individuals convicted of sexual offenses, 17 individuals started sexually offending when younger than 18 years, 16 started between the ages of 18 and 24 years, three between the ages of 24 and 30 years, nine between the ages of 31 and 40 years, and five when older than 40 years. It was concluded that these periods can be attributed to the emergence of sexual exploration and peer activities in adolescence, as well as changes in an individual’s family, work, and social circumstance. Although this study has a relatively small sample size, their conclusions help explain why people may begin offending at different ages.

In terms of the early onset of sexually deviant behaviors, Marshall et al. (1991)**** found that early onset is one of the strongest predictors of deviant behavior later in life. However, McKillop et al. (2015)***** found that in their sample of 306 adult males convicted of sexual offenses (aged 20–84 years), 8% of the sample started to sexually offend when older than 50 years. Should their sample be representative of the wider sexual offending population, this is not an insignificant proportion starting their sexual criminal career at a later stage of life.

With regard to the type of sexual offense, those convicted of child sex offenses have an older age of onset when compared with those convicted of adult sex offenses. For instance, Harris (2012)****, using a sample of 751 men convicted of sexual offenses and evaluated at the Massachusetts Treatment Center for Sexually Dangerous Persons between 1959 and 1991, found that individuals whose first recorded offense was for a sexual offense against a child had the latest onset of age (25.48 years old). In addition, Woodworth et al. (2013)***** found that the 139 high-risk individuals convicted of both adult and child sexual offenses (aged 19–77 years) who participated in their study had their first conviction at a much younger age (*M* = 23 years), compared with those who exclusively offended against children (*M* = 30 years). It was reported that the age of the first adult sexual offense ranged from 18 to 55 years (*M* age = 25.95 years, *SD* = 7.51), and the age of the first nonsexual offense ranged from 11 to 53 years (*M* age = 17.91 years, *SD* = 5.55). Moreover, Smallbone and Wortley (2004)*** reported that individuals convicted of extrafamilial offenses had a significantly earlier onset of sexual offending (*M* age = 28.9 years, *SD =* 11.40) compared with those convicted of intrafamilial offenses (*M* age = 34.2 years, *SD* = 10.43). Individuals convicted of intrafamilial offenses tended to be older (*M* age = 40.1 years, *SD* = 11.70) when convicted of a child sexual offense, compared with individuals convicted of extrafamilial offenses (*M* age = 34.0 years, *SD* = 13.16) or those convicted of both types of offenses (*M* age = 35.3 years, *SD* = 14.57). An explanation for the age difference can be inferred from a study by Mathesius and Lussier (2014)***** who found that men who targeted stranger victims exhibited an earlier actual onset compared with those offending within their biological family. The authors concluded that the differences in onset age might reflect situational factors, such as intimate relationships and access to children/victims. Moreover, disclosure should also be considered as a factor, as many cases of sexual assault and child sexual abuse largely go unreported.

In terms of the onset of general offending and criminal activity, Smallbone and Wortley (2004)*** found that approximately 80% of their sample were first convicted of a nonsexual offense. Seven studies found that males who were convicted of prior nonsexual offenses had a much earlier age of onset than those who had a first conviction of a sexual offense. Harris (2012)**** reported that if an individual started their criminal career with property crime (such as burglary or theft), their age at conviction for these crimes was significantly younger (*M* age = 15.91 years, *SD* = 5.16) than that at their first charge of violence (*M* age = 19.15 years, *SD* = 5.66) and sexual (*M* age = 23.87 years, *SD* = 8.99) offenses. Similar trends were found by Cann et al. (2007)****, in their sample of men convicted of crossover offenses, that men, on average, were older (*M* age = 37.1 years) when they began sexually offending than when offending in general (*M* age = 29.6 years). Moreover, Redondo et al. (2007)*** found that, of the sample of 123 individuals convicted of sexual offenses, those who recidivated (in this case, of general crimes) were significantly younger (on average, 25.2 years old) compared with nonrecidivists (on average, 34.3 years old) when committing their first sexual (convicted) offense. Furthermore, individuals convicted of sexual offenses released at a younger age were more likely to be general criminals, whereas those released at an older age were more likely to be “sexual offense specialists” (Thornton, 2006)*****.

It should be noted, however, that the age of onset of offending behavior may differ from the age at first conviction. Mathesius and Lussier (2014)***** noted that, on average, there was a 7.5-year age gap between the actual onset of sexual offending (offender’s age at the time of their first sexual offense) and the official onset of sexual offending (age when convicted and incarcerated for a sexual offense), in their sample of 332 incarcerated individuals convicted of sexual offenses. As stated above, the mean age of actual onset of sexual offending was 32.1 years (*SD* = 8.8), and the mean age of official onset of sexual offending was 39.6 years (*SD* = 12.2). The authors suggested that this gap is significant because “it allows offenders to remain at-risk of offending including sexual offenses; it increases the difficulty in obtaining forensic evidence for the initial conviction(s), and; it may contribute to offenders’ lowering their perception about the risk of sex offending” (Mathesius and Lussier, 2014, p. 140)*****. However, it is difficult to ascertain a “true” age of onset, given the reliance on the accuracy of data, and the legitimacy of disclosure and honesty of the individuals convicted of sexual offending.

#### Characteristics of offenders convicted of sexual offenses and the offense

Forty-three studies reported on the characteristics of individuals convicted of offenses and offense characteristics, including studies from Canada (*n* = 15), the United States (*n* = 9), the United Kingdom (*n* = 10), Australia (*n* = 2), Austria (*n* = 1), Brazil (*n* = 1), France (*n* = 1), Germany (*n* = 1), Italy (*n* = 1), New Zealand (*n* = 1), and Spain (*n* = 1). As shown in Table 1, only seven studies were deemed “lower quality” (rated one or two stars), and 22 studies were deemed “higher quality” (rated four or five stars).

#### Characteristics of individuals convicted of sexual offenses

Thirteen studies examined the characteristics of OSOs (typically categorized as individuals older than 60 years convicted of sexual offenses) or compared characteristics between the age groups. However, it must be noted that some studies investigating the OSO population did not use a comparison group. Collectively, the studies found that OSOs tended to be White, unemployed or retired at the time of offense, and have a history of steady and continuous employment (more recently employed as a car or truck driver and potentially have been in the military), and tended to have a higher educational level (e.g., Fazel et al., 2002***; Rayel, 2000; Tsagaris et al., 2017**).

In contrast to studies characterizing OSOs to be unemployed or retired at the time of their offending, two studies investigated professionals (e.g., physicians, teachers) who were convicted of sexual offenses. Dehlendorf and Wolfe (1998)**** found that physicians who were disciplined for sex-related offenses were, on average, older than the national physician population; among all physicians, 34.5% were aged between 45 and 64 years, whereas 58.1% of disciplined physicians were in this age group. However, there was no significant difference between physicians disciplined for sex-related offenses and the national physician population, for those older than 64 years. In addition, Sullivan and Beech (2004*****) found that this group tended to be older and more intelligent when compared with the broad population of individuals convicted of sexual offenses.

In terms of marital status, in comparison with OSOs, Brouillette-Alarie and Proulx (2013)***** reported that younger individuals convicted of sexual offenses were less likely to be in long-term relationships or have children. This would limit their access to intrafamilial victims and would provide one explanation as to why younger men tend to have extrafamilial and adult victims, compared with OSOs, who are more likely to have had long-term relationships and be married and have access to such victims. Conversely, Mathesius and Lussier (2014)***** suggested the typical adult male convicted for the first time for a sexual offense is in his late 30s at the start of his sentence has limited education and unemployed at the time of the sexual offense, and may or may not have a criminal record for a nonsex offense. Such men are more likely to have offended against females, have on average two victims, have offended approximately 10 times, usually within their familial environment (i.e., a family member), and were likely to be married or in a common law marriage. Clark and Mezey (1997)**** found that 77% of their sample were unmarried, though all their participants had been married at some point in their life. This was reflective of Rayel (2000)**, who found that in their sample of individuals older than 55 years convicted of sexual offenses, 86% were unmarried at the time of admission into the secure facility from which they were recruited. These two studies also found a history of sexual or physical abuse in childhood or adulthood was low for the OSO population, with Clark and Mezey (1997)**** reporting 8% had experienced such abuse, and 14% in Rayel (2000)**.

Regarding personality and physical and mental health of OSOs, the findings provided a mixed picture. Four studies reported that aside from low rates of depression, there is no evidence of other mental illnesses within this population (Clark & Mezey, 1997****). However, in one study (Fazel et al., 2002***), it was identified that older individuals convicted of a sexual offense were more likely to have dementia and have a previous psychiatric history, compared with younger persons convicted of a sexual offense. In addition, Fazel et al. (2002)*** found that OSOs were less likely to be substance abusers, both for drugs and alcohol, compared with individuals not convicted of sexual offenses.

Two studies (Harkins et al., 2012****; Marshall, 2010****) found that when compared with their younger counterparts, OSOs were more likely to score lower on the Psychopathy Checklist-Revised (PCL-R) than younger individuals convicted of sexual offenses. In addition, Marshall (2010)**** reported that OSOs scored lower on the PCL-R Factor 2, demonstrating lower criminal lifestyle. It was also found that OSOs scored lower on the measure for treatment needs and had a greater number of victims. A significant statistical difference was found in phallometric assessments, evidencing that OSOs had more sexually deviant interests than younger persons convicted of sexual offenses, a finding that was also supported by Brouillette-Alarie and Proulx (2013)*****. Moreover, among OSOs, there were no differences in the level of deviant sexual interest between individuals convicted of first-time, recidivist, and nonrecent offenses. In their study interviewing professionals (e.g., offender managers), Bows and Westmarland (2018)***** found that OSOs had a variety of issues concerning their mobility and physical health, as well as mental health and sexual health (the latter finding contradicting previous findings). For instance, this group of males tended to experience erectile dysfunction, which can often lead to feelings of frustration and therefore, lead these men to actively seek children to fantasize about to achieve an erection.

In interviews with OSOs, Mann (2012)***** found that this population tended to justify their sexual offending behaviors, or avoid taking responsibility, by blaming external factors such as marital issues or alcohol abuse. Moreover, they tended to deny having caused any injury to their victims, and blamed society for negatively labeling them a “sex offender.” These attitudes, combined with being professionals and having a higher level of intelligence compared with nonprofessionals, led to the participants exhibiting arrogance and dismissive attitudes, as well as being self-assured and proud of their actions, according to Mann (2012)*****. However, negative labeling of individuals convicted of sexual offenses should be avoided across all the age bands, particularly within treatment settings (Willis & Ward, 2011****). In addition, Mann (2012)***** found that this group adapted well to the prison environment and were generally able to build strong relationships, contrary to previous reports (e.g., Prison Reform Trust and The Centre for Policy on Ageing, 2003, as cited in Mann, 2012) documenting isolation and loneliness, and a hostile and inadequate environment.

#### Offense characteristics

Studies found that the majority of OSOs were either related to (e.g., father, stepfather, or grandfather) or an acquaintance of their victim, had a greater number of victims and sex crimes, and were more likely to offend against children than adults. It was widely found (e.g., Firestone et al., 2000****; Langevin & Curnoe, 2008****) that OSOs were more likely to be convicted of incest offenses or child sexual offenses, with rapists being younger and underrepresented within this population of OSOs (Harry et al., 1993***). Moreover, one reason for OSOs to be convicted of child sexual offenses could be that their victims are weaker and easier to overpower and manipulate, as well as the OSOs themselves having less strength (Hanson, 2002); Poortinga et al. (2007)** suggested that OSOs experienced difficulties in overpowering victims older than 12 years. Such patterns could potentially also be attributed to the accessibility of victims.

Although the studies have small samples, only two studies explored the sexual abuse of elderly victims (Browne et al., 2018****; Jeary, 2005****). Browne et al. (2018)**** examined the differences between those who sexually offended against older women and those who sexually offended against children. In their sample of 28 males found that those who were convicted of sexual offenses against a child were significantly older than those who sexually offended against older adults, indicating that a young age of the offender may be an aggravating risk factor for this type of offense.

### Aging and Risk of Reoffending

#### Relationship between aging and reoffending

A vast amount of research has examined the effects of aging on the risk of future reoffending. Perhaps unsurprisingly, all the studies examining aging and reoffending (*n* = 44) included in this review demonstrated that as age increases, the likelihood of reoffending decreases (e.g., Eher et al., 2016; Hanson, 2002, 2006). This trend is evident cross-culturally and using different risk assessment instruments (e.g., Static-99, Risk Matrix 2000, RRASOR) and cohorts. Studies pertaining to risk of reoffending were conducted in Canada (*n* = 19), the United States (*n* = 10), the United Kingdom (*n* = 3), Sweden (*n* = 3), New Zealand (*n* = 3), Austria (*n* = 3), Australia (*n* = 1), Italy (*n* = 1), and Spain (*n* = 1). The vast majority of the studies were deemed “high quality,” with 30 studies rated as four or five stars, with no studies rated as one or two (see Table 1).

Hanson (2002) stipulated that there are two peak risk periods for reoffending: (a) during adolescence and early adulthood and (b) during the late 30s. Older (i.e., older than 50 years) men exhibit a lower static risk, despite having more time to accumulate a criminal history compared with younger men (Helmus et al., 2012). All the studies examining age and risk found that younger age bands (e.g., 18–24) indicated a higher risk group, compared with over 39 age bands that exhibit a lower risk at the age of release (e.g., Craig, 2011****; Hanson, 2006; Looman & Abracen, 2010*****; Nicholaichuk et al., 2014*****). For instance, Fazel et al. (2006)*****, in their sample of 1,303 adult males convicted of sexual offenses and released from prison in Sweden between 1993 and 1997, reported that individuals older than 55 years sexually reoffended at a rate of 6.1%, compared with those younger than 25 years, who sexually reoffended at a rate of 10.7%. In addition, Fazel et al. (2006)***** found that having a stranger victim was a strong risk factor of reoffending for individuals aged 55 years and older, and 26.1% of their sample aged over 55 years reoffended violently.

However, a debate concerning the rate of decline was evident from the included studies. For example, Hanson’s (2002) observations from his well-supported meta-analysis demonstrated a clear linear decline was found between aging and reoffending for men convicted of rape and incest, and a curvilinear relationship was evident for men convicted of extrafamilial child sexual offenses. For the latter group, the highest peak was between the ages of 25 and 35 years, compared with the rapist and incest groups who exhibited higher recidivism rates between the ages of 18 and 24 years. Prentky and Lee (2007)***** also observed a similar pattern; in a sample of 136 “rapists” and 115 “child molesters,” the rates of sexual reoffending for “rapists” remained stable until the end of the 30s at around 28%, which then dropped to approximately 22% in the 40s, continuing to steadily decline thereafter. Moreover, there were no detected reoffenses for the oldest group (older than 60 years), though the sample size for this group was small. For the “child molesters,” a quadratic, rather than linear, pattern was found. Those aged between 18 and 29 years had the lowest overall sexual reoffense rate than the “rapists” (21.1%). This rapidly increased to 41.5% for those released in their 30s, thereafter steadily declining, dropping to 35.75% for those released in their 40s, 23.15% in their 50s, and 16.7% in their 60s. These findings reflect a similar pattern that was observed in Hanson (2002), though the decline started in the 40s rather than 50s. Moreover, Rettenberger, Birken, Turner and Eher (2015)*****, in a sample of 836 individuals convicted of sexual offenses (including child and adult offenses), found a cubic relationship between age at release and recidivism, with two bends in the trend; a decrease between the first and second age bands was observed, followed by a vast increase between the second and third age groups, with recidivism rates dropping in the last age band. In terms of violence recidivism, the total sample and both subgroups (i.e., “rapists” and “child molesters”) demonstrated a consistent decrease in the proportion of recidivists across the four age bands, with a stronger aging effect for the “rapist” subgroup when compared with the “child molesters.” However, this effect was not evident between age and sexual recidivism. As such, Rettenberger et al. (2015)***** concluded that the aging effect (i.e., the older the man, the lower the recidivism risk for sexual violence) could not be supported. Similarly, Thornton (2006)***** observed a cubic trend, with a change in the declining slope at two age points; between ages 18 and 25 years, there was a high sexual recidivism rate, which rapidly decreased from 80% to 50% for individuals released after that age. There was no subsequent decline until the age of 60 years, when the rate declined again by approximately 40%.

A number of explanations for the declining pattern have been suggested. According to the studies using phallometric assessments, male sexual arousability peaks in postpubescence and decreases as age increases (Blanchard & Barbaree, 2005***). This supports the findings of Barbaree et al. (2003)****, who demonstrated that the decline in arousability began in adolescence, with the steepest rate of decline found between adolescence and the age of 30 years, and a gradual decline thereafter. As such, the authors concluded that the decline in sexual arousal could be one reason as to why sexual recidivism declines with age. In addition, in a study examining desistance from sexual crime, participants reported they would not reoffend because they were too old, too tired, and no longer had “it in them” (Harris, 2016, p. 1724*****). Hanson (2002) attributed the decline to three factors: self-control (i.e., self-control increases as age increases), opportunity (i.e., the opportunity for sexually offending decreases with age for “rapists,” as people often associate with other individuals of their own age, and most rape victims are young women who are known to the perpetrator), and sexual deviance (i.e., motivation for committing sexual offenses, though the author noted that sexual deviancy is not the only contributing factor). However, the opportunity to offend is seemingly not the case for individuals convicted of child sex offenses, as more opportunities for offending can arise (e.g., having grandchildren).

#### Age as a variable in risk assessment tools

Studies have reported that the “age-at-release” effect was effective for actuarial assessments (e.g., Barbaree et al., 2003****; Eher et al., 2016; Wakeling et al., 2011*****). This effect supports a number of actuarial risk assessment instruments (e.g., Static-99, RRASOR, SORAG, and the Risk Matrix 2000), which account for this pattern in their age variables (Craig, 2011****; Hanson, 2006). Thus, this apparent pattern evidences and supports the age-crime trend (i.e., as age increases, offending rates decrease).

However, researchers have questioned the applicability of risk assessment tools for different age groups (Fazel et al., 2006*****). Helmus et al. (2012), in their meta-analysis, found that the weightings for “age at release” items were overestimated for OSOs. This was echoed by Lussier and Healey (2009)*****, who suggested that consideration needs to be paid to the base rates of reoffending for age, and improvement to predictive accuracy could be made if the age effect was taken into account. Therefore, such researchers have proposed that rather than using the “age at release” variable, risk assessment instruments should use historical or unchanging factors (for instance, age of onset of criminal activity). For example, Harris and Rice (2007)***** argued that “age at release” did not provide incremental predictive validity over actuarial risk assessment scores, and therefore, age of onset is a better risk marker (i.e., individuals who have an earlier onset of offending display an increased risk of reoffending). However, they acknowledged that “age of onset” and “age at release” were strongly related, as those who start offending earlier were more likely to be released younger than those who start offending later.

#### OSOs and risk

Two studies (rated four and five stars) specifically examined the role of risk within the OSO population. Marshall (2010)**** found that first-time OSOs were at greater risk for recidivism compared with older men convicted of nonrecent sexual offenses (i.e., those who ceased their offending behavior without receiving any form of intervention), as they had recently committed their offenses. The men convicted of nonrecent sexual offenses also showed an ability to stop and manage their sexual behavior, which suggests they were at a lower risk of sexual recidivism. In addition, it was found that OSOs were more sexually deviant than younger individuals convicted of sexual offenses, but there were no differences in deviant sexual interest found between men convicted of first-time, recidivist, and nonrecent offenses. Marshall therefore questioned whether treating deviant sexual arousal was useful for this population; if deviant sexual interests perpetuate sexual offending, it can be assumed that nonrecent OSOs would have reoffended since their last known offense and first-time OSOs would have offended at a much earlier opportunity. It was therefore theorized that the opportunity for offending may be a significant contributing factor for sexually offending later in life (Marshall, 2010****), supporting Hanson (2002).

Finally, Bows and Westmarland (2018)***** interviewed professionals (e.g., offender managers) to examine practices in relation to managing the risk posed by OSOs in the community, with care or support needs. One of the main challenges for managing OSOs was their “trustworthiness.” It was found that though the majority of the OSOs on the participants’ caseloads were assessed as being low to medium risk, one participant reported that half of the OSOs they managed were considered high to very high risk due to their trustworthiness; it was perceived that OSOs were more likely to exploit their needs and their portrayal of being unsuspecting. Moreover, questions regarding three risk issues were identified: (a) managing the risk for individuals diagnosed with dementia, as any implemented conditions or restrictions, or skills/lessons taught in treatment, may be forgotten; (b) managing risk of OSOs who are housed in care homes as, for instance, grandchildren may frequently visit their relatives housed in the same facility; and (c) how their care needs will be met in the community (Bows & Westmarland, 2018)*****.

### Treatment

Only three studies examined the role of age in treatment, with all three studies being conducted in Canada. It can be questioned whether OSOs can benefit from completing treatment programs and interventions, with two of the studies finding no evidence of a link between age, risk, and treatment completion to outcome (Langevin, 2006*****; Olver et al., 2012***). Indeed, Olver et al. (2012)*** sought to examine the outcomes of treatment on OSOs, given they were more likely to be lower risk. In general, it was found that the treated cohort of those convicted of sexual offenses had a significantly lower risk compared with those who remained untreated. Again, in support of the previous risk and recidivism research findings, younger men (those younger than 50 years) reoffended at higher rates than OSOs (those older than 50 years), irrespective of treatment condition (i.e., whether they attended treatment or not). These findings did not suggest a three-way interaction of age, risk, and treatment completion to outcome; however, it appeared that the younger individuals gained more from treatment than the OSOs, though this could be due to OSOs being a lower risk group, and that individuals wanting and attending treatment tended to be younger.

Marshall (2010)**** determined that the type of offense for which men had been convicted (i.e., recidivist, first time, and nonrecent) should first be identified to match the appropriate treatment level to individual OSOs. For instance, recidivist OSOs should participate in a higher intensity treatment than first-time or nonrecent OSOs, as recidivists demonstrate a lack of control over their sexual behavior. In addition, it was proposed that first-time and recidivist OSOs were at greater risk of recidivism than nonrecent OSOs, as they went against the trend of the age-effect, having committed their offenses more recently. Moreover, nonrecent OSOs were able to desist from their sexual offending behavior without intervention, further suggesting that (a) nonrecent OSOs are at a lower risk of recidivism or (b) in accordance with the age-crime curve, the individuals had, essentially, grown out of offending. However, as Marshall (2010)**** stated, OSOs could be recidivists who “represent the most deviant and persistent sexual offenders” (p.63). It was therefore concluded that an exploration into first-time (factors responsible for onset of offending), nonrecent (factors associated with cessation of offending in absence of legal intervention), and recidivist (factors for maintenance of sexually offensive behavior after judicial sanction) OSOs is essential for discovering factors associated with sexual offending and informing treatment facilitators and researchers.

## Discussion and Conclusion

The aims of this systematic review were to specify the current knowledge of characteristics in OSOs, and to explore the similarities and differences among adult individuals convicted of sexual offenses across the lifespan. The initial number of hits was 10,680. After employing the inclusion/exclusion criteria on the titles and abstracts, the full texts of 788 studies were reviewed and subjected to the inclusion/exclusion criteria, which resulted in 100 studies that were included in this review. The “MMAT” (Hong et al., 2018) was used to appraise and describe the methodological quality of the studies and to assess the risk of bias. However, meta-analyses could not be assessed using this tool. To present and summarize the results in addition to the MMAT ratings, the first two authors qualitatively weighed the studies and prioritized quantitative studies on representative samples and meta-analyses, followed by studies which were given lower weights (i.e., qualitative studies or quantitative studies with restrictive sample size and scope).

The studies included in this review considered a broad range of areas including the age of onset of criminal and sexual offense behaviors, age similarities and differences in the characteristics of individuals convicted of sexual offenses and the offense; explored the risk levels across the age bands and the role of age and aging in assessing risk; and finally examined treatment suitability for OSOs. Although this review brings together and examines a large number of studies (*n* = 100) pertaining to OSOs and aging in the population of persons convicted of sexual offenses, it is evident there are still gaps in our knowledge and understanding of OSOs. However, the presented findings from this review do provide a good starting point for future investigation which is essential for identifying the risk and treatment needs of for this population.

It was apparent from the studies that the peak adult age of onset of sexual offending behaviors ranged from mid-20s to mid-30s, with a clear two distinct populations based on the age of onset: (a) during adolescence, but stop offending going into adulthood, and (b) most adults offenders start to offend in their 30s (Smallbone et al., 2008). However, according to McKillop et al. (2015), 8% of their sample started to sexually offend over the age of 50 years. However, there is a clear gap in our knowledge concerning the actual prevalence rate of OSOs and the proportion of this population who start to offend at a later stage of life. From the presented research, it is also unclear why these individuals start to offend at a later stage of life and the contributing factors that perpetuate the offending. This could have an important impact when assessing risk, and also on prevention strategies within specific populations (e.g., in relation to grandchildren).

Research that aimed to identify specific characteristics of OSOs found that they tended to be unmarried; unemployed or retired at the time of offense; more highly educated; scoring lower on the PCL-R; and more likely to sexually offend against children who were known to them (e.g., a family member or an acquaintance). However, this only provides a superficial overview of the characteristics of this population. For instance, it is not clear what the mental health needs are for this population (e.g., dementia and cognitive deterioration) and future studies should focus more clearly on the mental health of this population. OSOs’ treatment needs have to be more clearly examined and identified. The limited available research seems to suggest that OSOs generally tend to be highly intelligent, have low psychopathy, low depression, and are potentially morally disengaged, with sexually distorted interests. Consistently with this, it would be pivotal to research and develop treatment and interventions addressing dynamic and changeable dimensions, such psychosocial factors.

What was clear in the findings was that as age increases, the risk of reoffending decreases, with younger age bands representing the highest risk of reoffending. This supports the widely accepted effect that age has on offending behaviors. However, first-time OSOs go against this trend and as such, more consideration is needed as to whether this group is actually low risk (or their risk is skewed in relation to the onset of sexual offending behavior), and risk assessment tools should be reviewed. It would be advisable to administer different tools depending on the type of OSOs (i.e., nonrecent, recidivist, or first time) being assessed, or different risk categories could be included depending on the age at release or age at first offense. In addition, first-time OSOs, as well as recidivist OSOs, can be perceived as being “more risky,” as these groups have most recently offended, and could potentially be more sexually deviant (Marshall, 2010). The examination of static factors other than age at release could potentially be more appropriate for this group. Further exploration is needed into the actual recidivism rates for first-time OSOs, as well as whether this group is more sexually deviant when compared with younger individuals convicted of sexual offenses.

Finally, the few studies that explored treatment and age identified that there was no evidence of a link between age, risk, and treatment completion to outcome. Previously, research had suggested that though OSOs may not benefit from attending treatment, they should still participate in programs. However, research (e.g., Lovins et al., 2009; Mailloux et al., 2003) has suggested that low-risk offenders should not be subjected to interventions or treatment and, instead, would benefit more from no intervention at all. Moreover, we still do not know what this population’s treatment needs are and it may be possible that there is no link because treatments are not actually addressing this population’s needs, as opposed to them being lower risk. Therefore, an examination of the effectiveness of risk assessment measures for first-time OSOs is essential, to identify whether such assessments accurately predict recidivism within this subgroup, assess whether first-time OSOs are more risky, and to readdress the level of intervention they should be provided.

The findings from this review (particularly pertaining to the onset of sexual offending behaviors) largely emulate the principles of the Motivation-Facilitation Model of Sexual Offending (MFM; Seto, 2019). As highlighted by Seto (2019), it is important to understand the factors that underlie sexual offending behaviors, to develop more effective prevention strategies, assessments, and interventions. Although we have a good understanding of the risk that individuals convicted of sexual offending pose of reoffending, little is known concerning the factors associated with the onset of sexual offending. The MFM identifies three primary sexual motivations: (a) paraphilias (particularly pedophilia and hebophilia, nonconsensual sexual sadism, and exhibitionism); (b) high sex drive (can become a motivation for sexual offending when the desire for sex overrides any inhibitions that prevent them from having sex with someone who is unable to consent to sex); and (c) intense mating effort (varies depending on individuals, and sensitive to age and environmental factors). In addition to the motivations, facilitation factors are factors that overcome any trait (e.g., self-regulation problems and hostile masculinity) or state (negative affect and alcohol use) inhibitions that prevent individuals from acting upon the motivations. Situational factors play a key role in an individual’s propensity to sexually offend; individuals who are strongly motivated to sexually offend are more likely to create opportunities to offend (Seto, 2019). Despite motivation and facilitation factors being in place, sexual offenses cannot occur without opportunities to act; situational factors, including access to vulnerable victims, location, and time, are all relevant in the act of sexual offending. Indeed, this is particularly relevant for OSOs, who have easy access to their victims and are more than likely to choose a vulnerable victim (e.g., their grandchildren). However, as highlighted by Seto (2019), the motivational factors can become less relevant with age; for example, the “high sex drive” motivation is incongruent with the general findings that suggest that sex drive declines with age. There are emerging themes in terms of the characteristics within the findings of this systematic review that demonstrated some key components of the MFM, but there is no great exploration into the relevant strait and trait dimensions as highlighted by Seto (2019), such as individual differences, self-control, and cognitive distortions (Ciardha & Ward, 2013; Szumski et al., 2018). Moreover, as reflected in Seto (2019), despite the ease of using recidivism data, longitudinal studies are necessitated to examine the factors that contribute to the onset of offending.

Although situational factors are important, a more multifactoral approach may be necessitated, such as the Integrated Theory of Sexual Offending (ITSO; Ward & Beech, 2016). The ITSO considers four factors that may affect sexual offending behaviors: biological factors, ecological niche factors, neuropsychological factors, and agency-level factors. Given we still do not have a good understanding of these factors in relations to OSOs, more research is needed exploring these to understand why OSOs start to offend at a later stage of life.

### Limitations

There were a number of limitations to this systematic literature review. First, the studies that were included in this review were all written in English; as such, any non-English publications that may have been relevant to this review were neglected. Moreover, the validity of the quality assessment that was implemented could be questioned. In accordance with the MMAT, the majority of the studies (included after the full texts were reviewed) were included in the final synthesis. Although the MMAT provides a good indication on the quality of a wide range of studies (include qualitative, quantitative, and mixed methods designs), its reliability and validity can be questioned and studies ascertaining such ratings need to be conducted.

In terms of the studies that were included in this review, it was difficult to ascertain whether factors were unique to the OSO population, as numerous studies did not use a comparison group (i.e., compared the OSOs’ findings with their younger counterparts). Furthermore, the use of standard deviation when reporting the age of the samples (and not reporting the age range) affected the decision as to whether a study should be included in the review. For studies where it was not possible to identify the age range, it was decided that these should be excluded. Therefore, it is possible that some studies were not included that may have been relevant. Finally, there was no standard definition for what is considered “older,” with findings being reported on OSOs aged 50 years and older or OSOs aged 60 years and older.

### Implications for Future Research and Clinical Practice

This review presents a comprehensive exploration into our current knowledge and understanding of OSOs, as well as aging in the population of individuals convicted of sexual offenses. It synthesizes what we currently know and identifies the future directions that should be undertaken to inform risk assessment and treatment interventions. The studies included in this review have built a strong foundation on which to further develop research examining this population. First, research should develop theory as to why OSOs convicted for the first time of a sexual offense started their offending behavior at a later stage of life, a population that is contrary to previous, well-established research findings (i.e., the age-crime effect). In addition, research needs to examine the needs of OSOs, including physical and mental health, to address whether such risk assessments and interventions meet their needs and to further adapt and develop these.

Future research should also explore the prevalence of OSOs, as well as the proportions of first-time OSOs, recidivist OSOs, and nonrecent OSOs, as currently these remain unclear. Furthermore, research should provide an in-depth exploration and comparison between “younger” and “older” individuals convicted of sexual offenses, to uncover the similarities and differences between the two populations and also to identify any unique factors between the two groups. Again, this will affect the delivery of risk assessment and interventions, and target prevention strategies. This would also add to the development of theory surrounding the population of OSOs, including why first-time OSOs start offending at a later stage of life, how and why nonrecent OSOs stopped offending without intervention, or potentially why nonrecent OSOs stopped and then restarted offending when older.

## Supplemental Material

sj-pdf-1-sax-10.1177_10790632211024244 – Supplemental material for Older Individuals Convicted of Sexual Offenses: A Literature Review Click here for additional data file. Supplemental material, sj-pdf-1-sax-10.1177_10790632211024244 for Older Individuals Convicted of Sexual Offenses: A Literature Review by Rebecca L. Crookes, Carlo Tramontano, Sarah J. Brown, Kate Walker and Hayley Wright in Sexual Abuse: A Journal of Research and Treatment
