# Supplementary material for: Older Individuals Convicted of Sexual Offenses: A Literature Review
Source: Sex Abuse. 2021 Jun 26;34(3):341–71. doi: 10.1177/10790632211024244 (PMC8905121; doi:10.1177/10790632211024244)
Supplement: sj-pdf-1-sax-10.1177_10790632211024244 – Supplemental material for Older Individuals Convicted of Sexual Offenses: A Literature Review [file sj-pdf-1-sax-10.1177_10790632211024244.pdf]

SUPPLEMENT SUMMARY TABLE FOR "OLDER INDIVIDUALS CONVICTED OF SEXUAL OFFENSES: A LITERATURE REVIEW"

Author(s)	Country	Aim(s) of the study	Design/method	Sample	Key findings	Theme
**Ackerman, Harris, Levenson & Zgoba (2011)** **** **[1]**	USA	The article sets forth a national profile of the registered sex offender (RSO) population	Quantitative Analysis of case file data	445,127 registered sex offenders Sourced from registries from 49 States	Mean age, accounting for invalid and unknown cases, was 44.8 (SD = 13.32) with a range of 12–99 (n = 449,534) This is interesting, as more people are places on registries for long durations (or life) with little attrition, the mean age will continue to grow older. This contradicts research indicating decline in risk with aging; sex offence recidivism is rare with advanced age.	Characteristics of Individuals Convicted of Sexual Offenses and the Offense
**Amirault & Lussier (2011)** ***** **[2]**	Canada	To revisit the association between past offending and recidivism in a sample of adult male sex offenders	Quantitative Analysis of case file data	553 convicted sex offenders	Offenses occurring in most recent observation period (between ages 30–35) were the most informative of future violent/ sexual offending. However, this was only true for prior violent/sexual charges, as prior nonsexual-nonviolent charges were not predictive of recidivism. Violent/sexual offending between ages 30 and 35 was the only offending time period associated with reoffending after adjusting for the offender's age-at-release	Aging and risk of offending

**Babchishin, Hanson & Blais (2016) ***** [3]**	Canada	[To examine] the extent to which two commonly used risk scales for sexual offenders (Static-99R and Static-2002R) predict violent and general recidivism, and whether it would be possible to improve predictive accuracy for these outcomes by revising their items	Quantitative Risk assessment data	3,536 adult male sex offenders from Canada, USA and Europe Average age = 39	The lowest score would be a first time offender released after the age of 60; the highest score would be an offender under the age of 35 with an extensive criminal history Age had a large correlation with the BARR-2002R and a smaller correlation with general criminality.	Aging and risk of offending
**Baltieri & Andrade (2008) **** [4]**	Brazil	To evaluate the role of drug consumption among sexual offenders against females	Quantitative Observational, retrospective and cross-sectional study	131 male convicted sexual offences against females	The sexual aggressors against women as a group were significantly younger than the offenders against girls and the offenders against pubertal females. There were no significant differences in terms of age between the aggressors against girls and the aggressors against pubertal females	Characteristics of Individuals Convicted of Sexual Offenses and the Offense

**Barbaree, Blanchard, & Langton (2003) **** [5]**	Canada	Study 1: examined the magnitude of erectile responses of sex offenders as a function of age<br>Study 2: address potential confound by comparing age-at-release cohorts using Kaplan-Meier survival analysis, which controls for time-at-risk.	Quantitative<br>Study 1: Experimental, Phallometric assessment<br>Study 2: Analysis of clinical files	Study 1: 1,413 male patients (mean = 37.17 years; SD = 13.32)<br>Study 2: 175 rapists, 155 child molesters, 93 familial offenders, and 45 mixed offenders<br>Average age 40. Age range 21 - 83	Study 1: Male libido highest in adolescence (peaks early 20s) and declines thereafter. Raises question whether this is due to a decline in testosterone or decreasing target tissue sensitivity?<br>Study 2: Recidivism decreased in a linear fashion over age-at-release. While age-at-release cohorts differed according to actuarial risk, the age-at-release effect was found to be significant when actuarial risk was controlled using Cox regression	Characteristics of Individuals Convicted of Sexual Offenses and the Offense

**Barbaree, Langton, Blanchard & Boer (2008)** ***** **[6]**	Canada	The Sexual Violence Risk-20 (SVR-20) was scored for each offender and the relationship between age-at-release and SVR-20 item and total scores was examined.	Quantitative Analysis of case file data	468 sex offenders 175 rapists; 155 child molesters, 93 familial offenders; 45 mixed offenders; 5 sexual offenders with adult male victims, and 3 offenders with non-contact sexual offenses	Significant difference among the mean ages-at release, released on average in the following rank order from youngest to oldest: rapists, mixed offenders, non-familial child molesters, and incest offenders. Rapists were released at a significantly younger age than the other groups, and mixed offenders were released at a significantly younger age than the incest offenders.<br>Risk factors prevalent in rapists and mixed offenders (e.g., antisocial behavior) more prevalent in younger offenders, whereas risk factors more prevalent in child molesters (e.g., paraphilia) more prevalent in older offenders.	Aging and risk of offending

**Barbaree, Langton, Blanchard & Cantor (2009) **** [7]**	Canada	The present study continues [the researchers'] exploration of the relationship between aging and actuarial risk in the prediction of recidivism in sex offenders	Quantitative Analysis of case file data	468 adult male sex offenders; 175 rapists, 155 child molesters, 93 familial offenders, 45 mixed offenders	Most actuarial items in sex offender assessment instruments were correlated with the age at release from custody. Items reflecting aspects of antisocial behavior were negatively correlated with age at release, whereas items reflecting sexual deviance were positively correlated.<br>Antisocial traits more prevalent among rapists who were released from custody at a younger age, whereas aspects of sexual deviance were more prevalent in child molesters who were released from custody at an older age.	Aging and risk of offending

**Beauegard, Deslauries-Varin & St-Yves (2010)** ***** **[8]**	Canada	To investigate the factors associated with decision making by sex offenders regarding confession during police interrogation; To identify the interactions among the factors that were significantly related to decision making regarding confession during police interrogation	Quantitative Survey data collected via interviews	624 convicted sex offenders Mean age = 39 (SD = 12.0)	Specialist Sex Offenders (SOs) younger than 40.5 years with an introverted personality most likely to confess; Specialist SOs older than 40.5 years with an extroverted personality less likely to confess More mature SOs seem to cope better with the unfamiliarity and demands of police interrogation As offenders grow older, they become increasingly resistant to interrogation.	Characteristics of Individuals Convicted of Sexual Offenses and the Offense
**Becerra-Garcia & Egan (2014)** **** **[9]**	Spain/UK	To study the changes in personality domains across the lifespan for a cohort of child molesters	Quantitative Personality domains measured using NEO-Five-Factor Inventory	169 male participants convicted of contact sexual offenses Age rage 19 - 77 (mean age = 44.4 years; SD = 12.29)	Neuroticism, Openness and Agreeableness in child molesters do not vary across lifespan, whereas E and C show similar changes to those of the general population.	Characteristics of Individuals Convicted of Sexual Offenses and the Offense

**Blanchard & Barbaree (2005) *** [10]**	Canada	To compare the sexual responsiveness of pedophiles, hebephiles, and teleiophiles, using a phallometric index of sexual arousability.	Quantitative Experiment; Phallometric measures	2028 male sex offenders<br>Age range: 13 - 79 (mean = 37.5; SD = 13.4)	Male sexual arousability highest in postpubescence and declines thereafter. The novel finding of the present study is the similarity of age-related decline in sexual responsiveness for pedophiles, hebephiles, and teleiophiles. If there are any differences in the aging rates in which these groups approach their preferred erotic objects in real-life situations (not addressed in this study), these differences are unlikely to be related to differential arousability	Characteristics of Individuals Convicted of Sexual Offenses and the Offense
**Blatier, Sellon, Gimenez & Paulicand (2016) ** [11]**	France	To determine whether these different cognitions would allow to distinguish the two groups of offenders, and whether they might enable us to differentiate sex offenders from incarcerated offenders for nonsexual crimes "other offenders"	Quantitative Experimental (completion of questionnaire and psychometric measures)	60 sex offenders<br>30 adults sex offenders and 30 child sex offenders<br>30 control participants<br>Mean age of adult SO = 42 (SD = 13.2). Mean age of child SO = 36.4 (SD = 12.2)	Age has a paradoxical effect: the older the individuals, the more they see the causes of their behaviour as controllable, whilst considering that the causes cannot be changed.<br>The more age increased, the more the scores on the Self-Esteem scale increased. The higher the age at first incarceration, the higher the respondents' self-esteem score.	Characteristics of Individuals Convicted of Sexual Offenses and the Offense

**Bourke, Ward & Rose (2012)***** [12]**	New Zealand	To investigate whether there are “expert” offenders within the child sexual offending domain who demonstrate high levels of competency in grooming techniques, target selection, interpretation and evaluation of environmental cues, and possess extensive offence scripts.	Qualitative<br>Semi-structured interviews	47 men convicted of sexual offences<br>Age range at time of index offense: 15-76 (mean = 36; SD = 14.05)<br>Age range at first sexual offense: 8-76 (mean = 29; SD = 15.67)	Individuals considered experts in the sample started their deviant sexual activities at an early age.<br>As they aged, their sexual preferences become fixed into a specific age range; their sexual preferences did not develop age appropriately as they matured.<br>Numerous salient behavioral traits were acquired by expert sex offenders in their early life and were utilized in the commission of their sexual offenses.<br>Thus, in later life they were able to manipulate others and viewed them as objects to be exploited to meet their own needs.	Characteristics of Individuals Convicted of Sexual Offenses and the Offense

**Bows & Westmarland (2016) ***** [13]**	UK	The aims of the study were to explore the number of sex offenders currently being managed, the issues that these offenders pose, how risk is managed in relation to offenders who have care/support needs, to highlight any best practice currently being followed and identify potential future issues	Qualitative<br>Face-to-face and telephone interviews	7 offender managers, including police/probation staff, or individuals working in the Public Protection Unit	Increasing number of Older Sex Offenders (OSOs), mostly involve the abuse of children and are historical in nature;<br>OSOs have needs in numerous areas, including housing and health care;<br>Challenges working with OSOs included perceived trustworthiness, finding suitable housing, deteriorating physical and mental health, and coming off the register;<br>Numerous issues in managing OSOs with care or support needs, particularly in those residing in care homes (e.g., grandchildren visiting residents in care homes and being in contact with vulnerable persons);<br>Contradicted research findings, reporting that offender managers deem OSOs to continuously pose a risk

**Broadhurst & Loh (2003)** **** **[14]**	Australia	To estimate the probabilities of re-arrest	Quantitative Analysis of police records	2,785 individuals were identified with at least one sex offence	Older non-aboriginal offenders (those over 30 years) had generally much lower probabilities of re-arrest for any and violent offences<br>Repeat older sex offenders had higher probabilities of re-arrest but again age had no influence on their times to re-arrest.<br>Very young offenders had probabilities of re-arrest for any offence of 0.89, while those over 50 years had much lower probabilities of 0.23.	Aging and risk of offending
**Brouillette-Alarie & Proulx (2013)** ***** **[15]**	Canada	To identify dimensions of the Static-99R, the most commonly used sex-offender actuarial scale, and to test their predictive validity	Quantitative Analysis of case file data	711 adult male sex offenders<br>352 sexual aggressors of children, 251 of women, and 90 mixed, 18 unknown<br>Age range = 18 - 77 years (mean = 40.85; SD = 12.08)	Younger SOs unlikely to be in long-term intimate relationships, to have children and therefore to have access to intrafamilial victims. This increases their chances of having extrafamilial/stranger victims, as they have limited access to intrafamilial ones. Older offenders who have had long-term relationships are more likely to have access to such victims.<br>Offenders with a sexually deviant criminal career tended to be older.	Characteristics of Individuals Convicted of Sexual Offenses and the Offense

**Browne, Hines & Tully (2018) **** [16]**	UK	To help fill the gap in the research into older adult sex abusers (OASAs) by comparing a group of OASAs with a group of child sex abusers (CSAs), using a sample of UK prisoners	Quantitative Analysis of psychometric measures	51 sexual offenders<br>Mean age of OASA = 37.25 (SD = 9.47)<br>Means age of CSAs = 44.78 (SD = 7.82)	There was a significant difference in age for OASA and CSA; the CSA group was significantly older than the OASA group; a young age may be an aggravating risk factor for this type of offence	Characteristics of Individuals Convicted of Sexual Offenses and the Offense
**Calkins Mercado, Alvarez & Levenson (2008) *** [17]**	USA	This research examined the perceived impact of community notification and residency restriction statutes among a sample of higher risk sex offenders in New Jersey	Questionnaire Self-report survey design	Survey distributed to 1,610 sex offenders listed on the New Jersey Sex Offender Internet Registry<br>137 surveys were completed	The majority (81%) of those who completed the questionnaire were between the ages of 25 and 64 years, while 11% of the respondents were aged 65 years or older;<br>4 of the participants were arrested for the first time over the age of 65.	Characteristics of Individuals Convicted of Sexual Offenses and the Offense

**Cann, Friendship & Gozna (2007) **** [18]**	UK	This study examined the proportion of sexual offenders in England and Wales who exhibited 'crossover' in their choice of victim, as defined by age, gender and relationship to the offender. It subsequently aimed to identify criminal and demographic variables predictive of crossover.	Quantitative Analysis of case file data (police records and criminal conviction histories)	1,345 adult male sexual offenders All received custodial sentences of four or more years	Offenders were, on average, older when they began sexually offending than compared to general offending. Crossover offenders had significantly more convictions for sexual offences and for any offence overall (including sexual). They were also significantly riskier in terms of sexual and violent recidivism, younger when first convicted of a sexual offence, and older when discharged from custody for their index offence.	Characteristics of Individuals Convicted of Sexual Offenses and the Offense
**Carabelleses, Candelli, Vinci, Tamma, & Catanesi (2012) * [19]**	Italy	The aim of this case report is to describe two cases of sexual abuse by elderly subjects for which the Judge commissioned an expert psychiatric–forensic opinion	Qualitative Case study	Two cases of elderly SOs; one in their 40s, and one in their 70s	Contrary to research findings, both cases featured forcible rape No evidence of mental illness Offences were planned in a rational, organised fashion No history of committing similar acts and had no history of homosexuality both had been married for many years before the death of their wives and had children, now adult	Characteristics of Individuals Convicted of Sexual Offenses and the Offense

**Castonguay, Proulx, Aubut, McKibben & Campbell (1993) *** [20]**	Canada	To determine whether some variables related to the patients or their offences could be related to the patients or their offences could be related with the magnitude of their penile responses.	Quantitative Experimental: Phallometric assessment	92 sex offenders<br>35 homosexual paedophiles, 21 heterosexual paedophiles, and 26 rapists<br>Age range: 16 - 61	As the age of the subject increased, observed magnitude of penile responses decreased. As such, this assessment more appropriate for younger subjects	Characteristics of Individuals Convicted of Sexual Offenses and the Offense

**Clark & Mezey (1997) **** [21]**	UK	To describe the characteristics of elderly Child Sex Offenders (CSOs) and their offences in order to consider any differences between them and their younger counterparts.	Quantitative Analysis of case file data	13 male CSOs aged over 65.<br>Age range: 65-89 (Mean age = 72.5)<br>Mean age at the onset of the most recent offences was 67.8 years	Relative family and social stability in early life and adulthood.<br>3 participants disrupted upbringing by loss events or multiple care-takers before age 11. 10 were married at the time of offences; all subjects had been married at some point. 12 gave a history of steady and continuous employment. 10 were retired at time of current offence and in 2 cases an onset of SO was closely related to retirement from full time work. 2 of the subjects had a history of excessive alcohol use, none admitted to illegal drug use. 8 had medical problems.<br>5 faced charges for SO of family members, 8 offended against kids outside of family but were acquainted with (i.e. neighbours).<br>9 offended against females, 2 against both sexes, and 2 against boys only. Victim age range 3-11.<br>Penetrative abuse occurred in eight cases; 5 involved fellatio, and one of the victims subjected to buggery. 10 of the subjects had been offending against the current victim for more than year, and for more than 10 years in four of these cases.	Characteristics of Individuals Convicted of Sexual Offenses and the Offense

**Cohen, Frenda, Mojtabai, Katsavdakis & Galynker (2007) ***** [22]**	USA	To examine whether sexual offenders against children have fewer characteristics associated with impulsive-aggression and more characteristics associated with aberrant sexual arousal than do adolescent and adult offenders	Quantitative Analysis of case file data (New York State Sex Offender registry)	837 sex offenders	Offenders against children (OPs) were more likely to be older and less likely to use either physical force or a weapon. More likely to offend against male victims or both genders than to offend against female victims only. Less likely to have sexual intercourse.<br>OPs were also more likely to be older (aged 26-39 vs <25), have a male victim, be known to the victim, and commit more than one act	Characteristics of Individuals Convicted of Sexual Offenses and the Offense
**Coxe & Holmes (2009) ***** [23]**	USA	To identify possible differences between high- and low-risk sex offenders	Quantitative Evaluation of Static-99, Abel Assessment, Raven's and MMPI-2 data	285 sex offenders on probation.<br>Sentences ranged from 2-10 years.	Average age of the low-risk group was found to be significantly lower than the high-risk group.	Aging and risk of offending

**Craig (2011)** **** **[24]**	UK	To examine sexual and violent reconviction in sexual and violent offenders and examine reconviction rates and levels of actuarial risk associated with age bands	Quantitative Analysis of case file data	131 offenders<br>85 SOs (mean age = 34.3, SD 9.3)<br>46 non-sexual violent offenders (mean age = 27.8, SD = 8.2).	Inverse linear relationship between age and rate of reconviction, with the youngest age band (≤24 years) presenting the greatest rate of reconviction and the oldest age band (≥45 years) presenting the lowest reconviction rate at the 5-year follow-up, except for sexual reconviction.<br>Plateau effect between the middle age group (35-44 years)<br>Significantly more younger offenders were categorized as high risk compared with 35-to-44 and ≥45 age offenders	Aging and risk of offending

**Curtice, Parker, Schembri Wismayer & Tomison (2003) *** [25]**	UK	(1) To generate data describing the characteristics of elderly referrals to a British regional medium secure unit (RSU); (2) To ascertain whether the complex needs of such patients were being suitably assessed from an 'holistic' perspective as recommended by the Reed Report (Department of Health and the Home Office, 1992), and to generate auditable guidelines and standards for use within the RSU for the assessment of future elderly offender referrals; (3) To provide data as a focus for initiating dialogue between local forensic and old age psychiatric services regarding the care of, and the development of services for, elderly offenders.	Quantitative Analysis of case file data	18 older sex offenders<br>Age range: 65 – 84 (mean = 69.5; SD = 4.4)	The spectrum of sexual offending included rape, unlawful sexual intercourse, incest and indecent exposure with indecent assault being the commonest sexual offence<br>All involved minors and all were related to recent offending rather than retrospective historical allegations<br>No mental disorder was found in 12/18 (67%) cases, and dementia was the most common diagnosis, present in 3/18 (17%) cases.<br>44% (8/18) had no previous forensic history but 39% (7/18) had previous convictions for sexual offences.	Characteristics of Individuals Convicted of Sexual Offenses and the Offense

**Dehlendorf & Wolfe (1998) **** [26]**	USA	To determine the frequency and severity of discipline against physicians who commit sex-related offences and to describe the characteristics of these physicians.	Quantitative Analysis of case file data	761 physicians disciplined for sex-related offences from 1981-1996	Physicians disciplined for sex-related offence were older than the national physician population. Among all physicians only 34.5% were between the ages 54 and 64, whereas 58.1% of disciplined physicians were in this age group.	Characteristics of Individuals Convicted of Sexual Offenses and the Offense
**Dickey, Nussbaum, Chevolleau & Davidson (2002) *** [27]**	Canada	[To investigate] the role of age in three categories of 168 offenders, including 53 rapists, 62 pedophiles, and 53 sadists.	Quantitative Analysis of case file data	168 male sex offenders Age range: 18-70 Classified into three groups: 18-25, 26-40, 40-70.	Older adult group were predominantly pedophiles (60%), followed by saidsts (30%) and rapists (10%). Findings consistent with hypothesis that the rapist group demonstrate greatest decline in sexual offending rates over age of 40. Decline could reflect decline in sex drive and physical ability.	Characteristics of Individuals Convicted of Sexual Offenses and the Offense

**Doyle, Ogloff & Thomas (2011) *** [28]**	Australia	The aim […] was to provide a comprehensive characterization of those offenders placed under post-sentence detention and supervision orders in Australia.	Quantitative Analysis of case file data	50 sex offenders<br>Age range: 20 - 74 (mean age = 44.4 years; SD = 13.29)	Onset of sexual offending for large proportion of the group was at a relatively young age (i.e., < 24 years)<br>Substantial variation in the chronicity of sample's sexual offending histories.<br>Early onset of general criminality, and committed a variety of non-sexual offences ranging in severity from breach and minor drug offences to serious crimes of violence	Characteristics of Individuals Convicted of Sexual Offenses and the Offense
**Eher et al (2016) *** [29]**	Austria	To evaluate whether sadism is indeed associated with higher rates of violent (including sexual) reoffending.	Quantitative Meta-analysis, and follow up study/analysis of case file data	Study 1: 2,169 male sex offenders sourced from 7 samples<br>Study 2: 768 male sex offenders	Age at release was inversely and significantly related to violent reoffending<br>Sadists in the sample tended to be older than the non-sadistic sex offenders at the time of their release from custody	Characteristics of Individuals Convicted of Sexual Offenses and the Offense<br>;<br>Aging and risk of offending

**English, Jones, Patrick & Pasini-Hill (2003) ***** [30]**	USA	This paper describes the need to incorporate information learned from the postconviction polygraph examination into intense treatment and criminal justice supervision	Quantitative Experiment	180 convicted sexual offenders (most convicted of crimes against children). 9.5% of the sample aged over 55 years	26% of the sample reported the onset of hands-on deviant behaviour when they were between the ages 5-8; 23% reported onset ages 9-11; 21% reported hands-on onset at 12 or 13, and 27% reported onset between 14 and 20. Early onset is one of the strongest predictors of "serious, long-term and frequent" deviant behaviour later in life	Characteristics of Individuals Convicted of Sexual Offenses and the Offense

**Fazel, Hope, O'Donnell, & Jacoby (2002) *** [32]**	UK	The study investigated the prevalence of psychiatric morbidity and personality disorders in elderly incarcerated sex offenders compared with elderly non-sex offenders.	Mixed methods Interviews and analysis of case file data	101 sex offenders and 102 non-sex offenders aged over 59 years. Age range = 60 - 88 (mean age = 65.9; SD = 4.7)	Sex offenders more likely to be white, unemployed at the time of their offence, most recently worked as a car or truck driver, and have a past psychiatric history recorded in their medical notes; Age of conviction for the index offence of the sex offenders was significantly older' No significant differences were found between the sex offenders and other offenders for diagnoses of psychotic illness, major depressive episode, organic disorder (dementia) and personality disorder. SOs less likely to be substance abusers; Most of the sex offenders were the father, stepfather, grandfather, or an acquaintance of their victim; and had committed crimes on several girls, and usually alone in the offender's home. There were no differences in the rates of psychiatric illness between these two groups apart from being assigned a diagnosis of personality disorder.	Characteristics of Individuals Convicted of Sexual Offenses and the Offense

**Fazel, O'Donnell, Hope, Gulati & Jacoby (2006) *** [31]**	UK	[To test] the hypothesis that frontal lobe changes are associated with sexual offending in older men	Quantitative Standardized questionnaire	50 sexual offenders and 50 non-sexual offenders	No evidence to suggest that sexual offending in older men is associated with frontal lobe dysfunction.	Characteristics of Individuals Convicted of Sexual Offenses and the Offense
**Fazel, Sjöstedt, Långström & Grann (2006) ***** [33]**	Sweden	To report rates of repeat offending by age and examine whether risk factors for violent recidivism are stable across age groups	Quantitative Analysis of case file data	1,303 adult male SOs released from prison in Sweden during 1993-1997	Those aged 55 and over sexually reoffended at a rate of 6.1%, compared with a rate of 10.7% for those aged under 25.<br>Having a stranger victim was a strong risk factor in those 55 and over.<br>26.1% (6/23) of older SOs reoffended violently (needs further research).<br>Rates of sexual and violent recidivism drop significantly by age, and in the older age band, all but one reconviction were for sexual offences.<br>Questions the applicability of risk assessments across all ages.	Aging and risk of offending

**Felson & Cundiff (2014) **** [34]**	USA	[…] examining age and gender patterns in a sample of sexual assaults and by comparisons of sexual assault with physical assault	Quantitative Analysis of case file data (from the National Incident-Based Reporting System)	Database contained 294,484 incidents of sexual assault involving a single victim and single offender	Older offenders were more likely than younger offenders to assault older females.<br>Older male offenders were more likely to commit sexual assault than physical assault<br>Older offenders have almost as strong a preference for adolescents and young adults as do younger offenders	Characteristics of Individuals Convicted of Sexual Offenses and the Offense
**Firestone, Bradford, Greenberg & Serran (2000) **** [35]**	Canada	To a) compare the prevalence of psychopathy and phallometric indexes of deviant sexual arousal and, b) study the relationship between phallometrically assessed deviant sexual arousal and psychopathy, as measured by the PCL-R, among a large sample of incest offenders, child molesters and rapists.	Quantitative Analysis of psychometric measures	156 incest offenders<br>260 extrafamilial child molesters<br>123 rapists	Incest offenders were found to be significantly older than the rapists and child molesters, and child molesters were significantly older than rapists<br>Incest perpetrators tend to be older as their sexual offenses are more opportunistic in nature. They do not offend until after they are married and have children.<br>Child molesters tend to be more sexually aroused to children than incest offenders, are more predatory in their offense patterns and often are younger when they start their sexual offenses.	Characteristics of Individuals Convicted of Sexual Offenses and the Offense

**Freedman (2012) *** [36]**	USA	To examine: (1) the association between recidivism and registration failures; and (2) the utility of static risk factors, including registration failures, for predicting recidivism	Quantitative Analysis of case file data	191 registered sex offenders<br>Mean age = 45.82, SD = 1.63	Age is negatively associated with recidivism, while previous convictions have a positive association	Aging and risk of offending
**Freeman (2007) ***** [37]**	USA	To compare the predictors of rearrest for rapists and child molesters on probation.	Quantitative Analysis of case file data (New York State Sex Offender registry and criminal history database)	5,331 males convicted of sexual offenses. Average sex offender was 35.95 at time of first sexual arrest and 35.57 (13.03) at time he was arrested for the registerable sexual offence, with a range between 15 - 85 years of age.	Offender's age at the time of the instant offence arrest is a significant variable<br>Each 1-year increase in age reduced the likelihood of being rearrested for a sexual offence by 0.3%<br>Each 1-year increase in age reduced the likelihood of a rapist being rearrested for a nonsexual offence by 1.8%, whereas it reduced the probability of a child molester being arrested for nonsexual offence by 0.8%.	Aging and risk of offending

**Fruehwald et al. (1998) ***** [38]**	Austria	[The researchers] analysed self-concepts of 53 long-term incarcerated sex offenders to research the correlation between concepts of self-esteem, assertiveness, feelings towards others and relationships and the degree of violence of the last offense and risk of re-offense	Quantitative Survey: Frankfurt Scales of Self-Concepts	53 sex offenders	Mean age of the offenders was 35.9 years (SD = 10.5, range 21-72), mean age at first conviction was 22.3 (SD=8.5, range 15-66).	Characteristics of Individuals Convicted of Sexual Offenses and the Offense
**Garombo et al. (2016) *** [39]**	Italy	[To evaluate whether] RM-2000 Italian validation provides an objective instrument of sexual static recidivism risk for the professional operators who work in this field.	Quantitative Risk assessment data (RM 2000)	308 male offenders Age range 19-85 (mean age = 49.3; ± 7.3 years)	Low risk offenders have a mean age of 53 years, medium risk a mean age of 48.3 years and high/very high risk a mean age of 39.1 years old. Statistically significant differences in age at administration with a decreasing gradient of mean age that is correlated with increasing the risk category	Aging and risk of offending
**Hanson & Bussiere (1998) [40]**	Canada	Study presents a quantitative review of the sexual offender literature	Quantitative Meta-analysis	Reviewed 61 studies totalling 28,972 SOs	Of the demographic variables, only age (young) and marital status (single) were related to sexual offence recidivism. Nonsexual, violent recidivists tended to be younger. General recidivists tended to be young	Aging and risk of offending

**Hanson (2002) [41]**	Canada	To examine the relationship between age and recidivism	Quantitative Meta-analysis	10 samples of adult sex offenders (total 4,673). Samples from Canada, US and UK	The recidivism rate declined steadily with age; Significantly higher recidivism rate for extrafamilial child molesters than rapists; Relationship with age was different for the rapists, extrafamilial child molesters, and incest offenders; The recidivism rate for rapists steadily decreased with age; Highest risk age period for extrafamilial child molesters was 25 - 35. Recidivism rate of extrafamilial child molesters showed little decline until after age 50; The recidivism rate for the incest offenders was generally low; Very few recidivists among the sexual offenders released after age 60; none of the incest offenders (n = 39) or rapists (n = 10) released after age 60 recidivated. Three factors to explain the recidivism trend: sexual deviancy (motivation), opportunity, and low self-control	Aging and risk of offending

**Hanson (2006) [42]**	Canada	To examine the combined effect of age and Static-99 scores on the prediction of sexual recidivism.	Quantitative Meta-analysis Evaluation of Static-99 data	Data sourced from 8 samples (combined size of 3,425 sexual offenders)	Average age at release was 37.9 (age range 18-85).<br>When controlling for Static-99 scores, the influence of age was curvilinear, with30 years being the age at greatest risk.<br>In all the analyses (with or without controlling for Static-99 risk factors), there was a steady decline in recidivism rates for offenders after the age of 40 years.<br>Older offenders had lower Static-99 scores than younger offenders<br>Offenders younger than 25 had the highest scores, followed by offenders aged 25 to 39, offenders in their forties, offenders in their fifties and offenders older than 60.<br>Of the 54 men released after the age of 70, there was only one sexual recidivist (a child molester with a Static-99 score of 4)	Aging and risk of offending

**Hanson, Harris, Letourneau, Helmus, & Thornton (2018) [43]**	Canada	To extend previous research on the declining risk of sexual recidivism over time (Hanson et al., 2014) by statistically modelling the effects of time sexual offense-free in the community, initial risk level, age, and subsequent nonsexual offending.	Quantitative Meta-analysis	Combined sample size of *N* = 7,000, from 20 samples	Age was not related to recidivism risk once Static-99R scores were entered. No interaction between age and time free; 'free effect' applied to sexual offenders of all ages. Though aging may explain some effects, the time free declines were larger than aging alone.	Aging and risk of offending
**Harkins, Beech & Thornton (2012) **** [44]**	USA	The current study was carried out as an attempt to examine the relationship between therapeutic climate of sexual offender treatment groups, risk level, psychopathy (in a treatment program that addresses responsivity as it is designed for psychopaths), and phase of treatment.	Quantitative; Analysis of psychometric and risk scales (GES; PDS; PCL-R; RM 2000)	137 sex offenders Mean age at testing = 42.8 (SD = 12.0)	Men in the lower PCL-R group being significantly older than the higher PCL-R group	Characteristics of Individuals Convicted of Sexual Offenses and the Offense

**Harris (2012)** **** **[45]**	USA	To determine whether participants could be differentiated on the basis of the nature of their first officially recorded offense.	Quantitative Analysis of archival files	751 men convicted of sexual offences<br>Age range: 14.82 - 70.85 years (mean = 29.78 years; SD = 9.94)<br>Rapists were youngest offender type, followed by mixed offenders, child molesters, then incest offenders	Age of onset differs across offense types. Offenders who were first convicted for a property offense had an early onset and committed largest number of offenses, over longest period of time.<br>Offenders with sexual onset were first charged after the age of 20, and had the lowest number of charges over shortest period of time.	Characteristics of Individuals Convicted of Sexual Offenses and the Offense
**Harris (2016)** ***** **[46]**	USA	[To] examine the narratives of 60 men interviewed in the community, who were incarcerated for sexual offenses and released	Qualitative Semi-structured interviews	60 men convicted of a sexual offense<br>88% identified as White.<br>Age range 24-73; average age 53.<br>Sentence length 4 months - 38 years	Desistance by age: offenders reported to have naturally matured out of crime. Reports not likely to reoffend because "too old and too tired". Desisters were youngest of all the styles. Criminal behaviours started in early adolescence. 'Rote' style of desistance: oldest participants, age ranging 29-79. Offenses child molestation and step-child incest. Most persistent histories of sexual offending, and seven participants reporting recidivism.	Aging and risk of offending

**Harris & Rice (2007) ***** [47]**	USA	[The researchers'] purpose is to provide nontechnical support for users of actuarial risk assessments.	N/A	N/A	‘Age at release’ did not provide incremental predictive validity over actuarial risk assessment scores, and therefore, age of onset is a better risk marker	Aging and risk of offending
**Harry, Pierson & Kuznetsov (1993) *** [48]**	USA	To examine a wide range of offender, victim and offence variable contained in a database derived from a large group of Missouri sex offenders	Quantitative; Analysis of case file data	808 sex offenders	Rapists tended to be younger	Characteristics of Individuals Convicted of Sexual Offenses and the Offense
**Helmus & Thornton (2016) [49]**	USA	To highlight concerns with the development of the MATS-1 and to validate the scale using 3,510 sex offenders from 14 unique samples	Quantitative Meta-analysis	3,510 sexual offenders sourced from 14 samples	The MATS-1 significantly underestimated recidivism for offenders in their 40s, as well as offenders age 60 and older.<br>For offenders age 60 and older, the MATS-1 significantly underpredicted recidivism by about half. In other words, the predicted recidivism rate for this group was half of the actual recidivism rate	Aging and risk of offending

**Helmus, Thornton, Hanson, & Babchishin (2012) [50]**	Canada	To examine whether Static-99 and Static-2002 adequately capture the relationship between age at release and recidivism (primarily sexual recidivism, although violent recidivism was also examined).	Quantitative Meta-analysis	Total of 8,390 sex offenders, from 24 separate samples	Age was negatively correlated with Static-99 and Static-2002 scores<br>Older offenders scored lower in static risk, despite having more time to accumulate a criminal history than younger offenders<br>Clear decline in recidivism among older sex offenders<br>Age is more strongly related to nonsexual violent recidivism than to sexual recidivism	Aging and risk of offending

**Janka, Gallasch-Nemitz, Biedermann & Dahle (2012)**** [51]**	Germany	[The researchers examined] the general predictive potential of offending behavior variables for heterogeneous sexual offender populations without further stratifications.	Quantitative Analysis of police records	682 male sex offenders<br>*n* = 321 convicted of sexual coercion or rape<br>*n* = 308 convicted of child sexual abuse<br>*n* = 53 convicted of both.<br>Age range at time of offense: 14 - 77 (mean = 35.28; SD = 11.77)	For middle-aged offenders (35–49 year olds), the variables 'Securing the crime scene', 'Making amends' and 'Avoiding/removing evidence' helped to improve prediction of sexual recidivism. These are risk-minimizing strategies, to avoid discovery during the offense and during later investigations<br>For the oldest group of offenders (≥50 year olds), the variables 'Cruising', 'Sexualized speech' and 'Avoiding/removing evidence' were significant. The latter can be interpreted as evidence of a more sophisticated offending behavior.	Characteristics of Individuals Convicted of Sexual Offenses and the Offense
**Jeary (2005) ***** [52]**	UK	The paper presents findings relating to the abusers/offenders and their victims, discussing the types of abuse, the circumstances, and the relationships between perpetrator and abused	Qualitative Documentary analysis, interviews, focus groups and transcription analysis	52 cases involving the victimisation of elderly people over the age of 60	The age range of offenders included was 16 – 70+ years at the time of conviction<br>Offenders aged 16 – 30 years at the time of conviction were responsible for two-thirds of the sexual assaults and killing and the rape/attempted rape offences in the study.<br>Two men sentenced for the first time when aged 60 + years	Characteristics of Individuals Convicted of Sexual Offenses and the Offense

**Langevin & Curnoe (2008)** **** **[53]**	Canada	In the following study, relative contributions of sexual offending and paraphilias versus substance abuse, endocrine disorders, head injuries, neurodevelopmental abnormalities, learning disorders, age, education, and IQ to neuropsychological impairment are examined to determine whether sex offender status is predicted by neuropsychological impairment when these confounding influences are considered.	Quantitative Analysis of HR battery	1,180 sex offenders and paraphilics, and 113 non-sex offender controls	The sample averaged 36.95 years of age at the time of their initial assessment (S.D. = 13.30; range, 12–84). The SOPs were significantly older than the controls (offenders of children tended to be older that the offenders of adults)	Characteristics of Individuals Convicted of Sexual Offenses and the Offense
**Langevin & Curnoe (2011)** ***** **[54]**	Canada	The present study examines the relationship of Hare's measure of psychopathy for adults (PCL-R), ADHD, brain dysfunction, and recidivism in a forensic sample of sexual and violent offenders.	Quantitative Analysis of case file data	1,695 adult male sexual, violent, and nonviolent offenders; 1,520 sex offenders and paraphilics 133 violent non-sex offenders 42 nonviolent non-sex offenders	The average involvement with the criminal justice system started at 26.77 years of age and spanned 16.25 years. The groups also differed significantly on age at first involvement with the law, with the Psychopathic Proper (PP) group youngest, the Borderline psychopathic group (BP) next oldest, and the non-psychopathic group (NP) oldest of all groups	Characteristics of Individuals Convicted of Sexual Offenses and the Offense

**Langevin & Curnoe (2012) *** [55]**	Canada	To extend the 2004 study from 1966 to 2009 to show that long-term follow up of untreated sex offenders will be associated with a significantly higher rate of offending than typically reported in short-term follow-up studies; and to examine a number of other factors that may influence criminal history measures	Quantitative Analysis of case file data	2,190 sex offenders Age range: 12 - 84 (mean age = 35.6 years; SD = 12.8)	Repeat offenders were statistically and significantly younger than the first-time offenders at the time of initial assessment and, over their life span, were studied longer. The mean span of criminal career for the present sample was more than 16 years and ranged up to 52 years, and cases were followed up on average for more than 21 years and up to 49 years.	Characteristics of Individuals Convicted of Sexual Offenses and the Offense
**Langevin (2006) ***** [56]**	Canada	[To examine] the desire for, acceptance of, and completion of treatment among sex offenders	Quantitative Medical and psychological files were assessed.	778 male sex offenders who were psychologically assessed between 1966 and 2003 Age range: 13 - 82 years (average age: 35.6 years; SD = 12.4)	Offenders wanting and attending treatment were significantly younger than were those not wanting it or attending; No significant difference in age of treatment completers versus non-completers	Treatment

**Levenson, Sandler & freeman (2012) ***** [57]**	USA	To describe the characteristics of a sample of sex offenders charged with failure to register (FTR) in New York State, compare the FTR and non-FTR groups on relevant risk factors, identify risk factors associated with failing to register, and investigate the relationship between registration noncompliance and both general and sexual rearrest	Quantitative Analysis of case file data	7055 adult male sex offenders	FTR offenders were younger at release<br>Odds of an offender being charged with FTR were significantly reduced by offender age at release (3% reduction for every year older the offender was at the time of his release)<br>Odds of an offender being rearrested for a nonsexual offense were significantly reduced by offender age at release (4% reduction for every year older the offender was at the time of his release).<br>Odds of an offender being rearrested for a sexual offense were significantly reduced only by offender age at release (a 3% reduction in odds for every year older the offender was at the time of his release	Aging and risk of offending

**Looman & Abracen (2010) ***** [58]**	Canada	The current study explored the performance of the Static-2002, RM 2000, RRASOR, and Static-99 in 510 sex offenders assessed or treated at the Regional Treatment Centre Sexual Offender Treatment Program.	Quantitative Analysis of risk assessment tools (Static-2002, RM 2000, RRASOR, and Static-99)	419 released sex offenders	Static 2002: Only 'Persistance of Sexual Offending' and 'Age at Release' were significant predictors of sexual recidivism<br>Men aged 25 - 35 were more than 1.5 times more likely to sexually reoffend than either the group aged 50 or over or the group aged 18 - 24. The men aged 35 - 50 were intermediate between the 25--35-year-olds and the men above 50 in terms of their risk for reoffence.<br>Rapists: 'Age at Release' and 'Persistence' subscales were significant predictors.<br>Child molesters: Age at Release was not a significant predictor	Aging and risk of offending

**Lussier & Healey (2009) ***** [59]**	Canada	This study explored the role of age at release on the risk of reoffending using a sample of sex offenders	Mixed methods Interviews and analysis of actuarial measures	553 adult males convicted of a sexual offense<br>Age at onset (official): 16 - 75 (mean = 30.45; SD = 13.29)<br>Age at release: 20 - 77 (mean = 42.62; SD = 12.07)	For every one-year increase of age at release, recidivism rate decreased by 3%.<br>The base rate of reoffending for age categories should be considered when determining criminal justice interventions.<br>Both actuarial risk and age at release independently and significantly helped to identify recidivists and non-recidivists.<br>The predictive accuracy of sex offenders' recidivism could be improved by taking into account the age effect	Aging and risk of offending
**Lussier & McCuish (2016) **** [60]**	Canada	The […] aims to clarify the role of the social environment in the context of much adversity to desist from crime	Quantitative Analysis of case file data	500 individuals convicted of a sex crime between 2003 and 2012	The likelihood of committing a crime decreases with age, even for sex offenders.<br>Age did not moderate the relationship between prosocial/antisocial peers and desistance/persistence; having delinquent friends was associated with reoffending for both the 20-year-old and the 40-year-old offender.	Aging and risk of offending

**Lussier, Deslauriers-Varin & Râtel (2010) ***** [62]**	Canada	[To provide] a preliminary descriptive profile of individuals having been issued an 810 recognizance order (i.e., peace bond).	Quantitative Analysis of sociodemographics, risk profiles and recidivism of offenders	59 offenders who were issued an 810.1 (at risk of sexual offense against a child) or an 810.2 (as risk of a personal injury offense) Age range 18 - 72 (mean = 44.2 years old)	Younger offenders more likely to recidivate. Results suggest that for every 1-year increase in the offender's age, the probability of general recidivism decreased by about 4%.	Aging and risk of offending
**Lussier, Gress, Deslauriers-Varin & Amirault (2014) *** [63]**	Canada	To evaluate the impact of the CHROME program for offenders under an 810.1 and 810.2 order (under 810 recognizance orders in the Criminal Code of Canada) on the time spent in the community without reoffending and the type of reoffending they committed	Quantitative Quasi-experimental design	269 sex offenders issued with an 810.1 or 810.2 order between Jan 2003 and Feb 2009 Age range: 15-80 (mean = 41.2; SD = 13.0)	Age found to be a protective factor when considering risk, and recidivists more likely to be younger. Community re-entry more challenging for younger sex offenders characterized by higher actuarial risk of reoffending.	Aging and risk of offending
**Lussier, Proulx & Leblanc (2005) ***** [61]**	Canada	[…] compared the relative utility of these three hypotheses in explaining criminal activity in adulthood of sexual aggressors of women	Quantitative Questionnaire administered by interviews	209 adult males Mean age = 33.5 years old (SD = 9.0)	Mean age of onset for a sexual crime in adulthood was 30.03 (SD = 8.12).	Characteristics of Individuals Convicted of Sexual Offenses and the Offense
**Malcolm, Andrews & Quinsey (1993) * [64]**	Canada	Investigated the discriminant and predictive validity of phallometric assessments of sex offenders sexual age and gender preferences	Quantitative Experiment	172 men convicted of sexual offences	Child molesters tended to be older	Characteristics of Individuals Convicted of Sexual Offenses and the Offense

**Mancini, Reckdenwald, Beauregard & Levenson (2014) ***** [65]**	Canada	[To] draw on the main tenets of the social learning perspective and life course theory to explore the impact of multiple forms of sex industry exposure on offenders' propensity to commit sexual crimes.	Quantitative Retrospective, longitudinal data	616 adult males convicted of a sexual offense	Sample had a relatively older age of onset.<br>Sex industry exposure (pornographic movies, magazines, and prostitution use), was linked with a corresponding lower age of onset<br>Age and having a child victim were associated with a later age of onset<br>Those who victimized strangers and males were younger when committing their first sex offense	Characteristics of Individuals Convicted of Sexual Offenses and the Offense

**Mann (2012)** ***** **[66]**	UK	This article examines how child sex offenders, the most vilified subsection of the prison population, survive, and observes the differing ways in which both acceptance of, and resistance to, the prison regime become manipulated actions executed by extremely knowledgeable agents.	Qualitative Interviews	40 prisoners aged 55 and over	Majority of aging CSOs appeared to enjoy friendship developed amongst these type of offenders;<br>Most appeared to be high-skilled actors when adapting to the new prison structures to suit their needs, may be due to many were professionals who were well-educated. This allows them to regain some power and manipulate the system to gain certain privileges.<br>Some found the experience frightening and isolating; less skilled when adapting to new structures and unable to manipulate the system. These individuals found prison life difficult.	Characteristics of Individuals Convicted of Sexual Offenses and the Offense

**Marshall (2010) ***** [67]**	Canada	This thesis examined the effect of age on sexually offensive behaviour	Quantitative Experimental; Phallometric assessment	802 male sexual offenders (phallometric testing)	Oldest and youngest offenders were more deviant than middle-aged sexual offenders and that the sexual interests of older offenders were more commonly for females; Subjects classified as Historical, first-time or recidivist sexual offenders; 55% of the sexual offenders age 60 years or more were found to have committed and ceased their offending at least seven years prior to incarceration; percentage of Recidivist offenders remained relatively consistent across age groups at about 20%; No difference was found in phallometrically assessed sexual deviance between the groups of older offenders	Characteristics of Individuals Convicted of Sexual Offenses and the Offense; Aging and risk of reoffending; Treatment

**Marshall, Barbaree & Eccles (1991) **** [68]**	Canada	To describe the self-reported onset of deviant fantasies, the frequency of offences, and the incidence of multiple paraphilias in child molesters	Mixed methods Interviews and psychometric data	129 child molesters 91 non-familial offenders and 38 incest offenders	Incest offenders are older, less deviant, and have fewer victims; 45 (35.7%) reported they had committed their first sexual assault against a child before they reached the age of 20; Only 7.9% of incest offenders committed their first offence while under 20 (individuals would not have had children before age of 20)	Characteristics of Individuals Convicted of Sexual Offenses and the Offense

**Mathesius & Lussier (2014)** ***** **[69]**	Canada	The current study investigates the covariates of age of onset (actual and official) and cost avoidance of sex offending in first-time convicted sex offenders	Quantitative Analysis of case file data	332 sexual offenders	Mean age of actual onset of sex offending is 32.1 years (SD = 8.8), ranges between 14.2 to 73.1 years of age.<br>Mean age of official onset of sex offending is 39.6 years ranging 18.7 - 75.2 years.<br>Offenders able to avoid a conviction, on average, for 7.5 years (SD = 9.3) after the start of their sex offending.<br>Sex offenders in this study start offending in their early thirties. Official data reveals that their first sex crime conviction occurs in late thirties;<br>Typical adult first-time convicted sex offender is in his late thirties at the start of his sentence, has limited education, is married or in a common-law relationship, and may or may not have a criminal record for a non-sex offence. often unemployed at the time of their sex offence, and have an average of two victims of who they offended against approximately ten times.	Characteristics of Individuals Convicted of Sexual Offenses and the Offense

**Mattek & Hanson (2018) * [70]**	Canada	To examine the life of an especially persistent and exceptionally older offender to determine the extent to which these characteristics apply.	Qualitative Case study	A case study of Atypical Offender (AO); man committed as a sexually violent person in his mid-90s	Typical protective factors were absent in this case; AO was in good health, both physically and mentally; denial of offending and non-compliance with treatment. Concluded that it is "not unreasonable to view older individuals who are exceptionally healthy as also having a higher risk of recidivism than same-aged but less healthy peers"	Characteristics of Individuals Convicted of Sexual Offenses and the Offense
**McKillop, Brown, Smallbone & Pritchard (2015) ***** [71]**	Australia	To investigate, in the adult group, at what age they began sexually abusing children; and in the adolescent group the number who persisted with sexually abusive behaviour into adulthood To compare the characteristics of adolescence-onset sexual abuse incidents with the characteristics of adulthood-onset sexual abuse incidents	Quantitative Self-reported and official data	306 adult males convicted of child sex offenses Age range: 20 - 84 (mean = 44.6; SD = 12.0)	The average age at the time of the first sexual abuse incident (onset) for the adulthood-onset group was 34.4 years (SD = 11.7, range = 18–79 years). The majority (66%) were aged between 25 and 49; 25% were aged between 18 and 24; and 8% were aged over 50. A small proportion (9%) of adult offender sample reported first sexually abusing a child in adolescence	Characteristics of Individuals Convicted of Sexual Offenses and the Offense

**Navarro & Jasinski (2015) ** [72]**	USA	To investigate whether online sexual offenders' demographic background characteristics—as well as factors indicative of motivation—vary across offense types	Quantitative Analysis of secondary case file data	Survey sent to 2,598 state, county, and local law enforcement agencies that inquired about cases related to child pornography and sexual offenses (as a result of an online interaction)<br>1,051 arrest cases of online sexual offenders	The risk of arrest for child pornography without online sexual exploitation of an identified victim (CP) vs arrest for solicitation of online undercover law enforcement that involved no identifiable victims (O-UC) increased for offenders between 40 and 49 years old and 50 years old or older;<br>The odds favoring arrest for CP over O-UC are higher for offenders 40 years old and older if law enforcement considered them sexually deviant in a way that did not involve children or if they had been previously violent	Characteristics of Individuals Convicted of Sexual Offenses and the Offense

**Nicholaichuk, Olver, Gu, & Wong (2014) ***** [73]**	Canada	To provide data with respect to the outcomes and characteristics of a cohort of offenders who were over 50 at age of release and examined the probability and severity of violent and sexual recidivism in this group.	Quantitative Analysis of case file data	2,158 male sex offenders<br>Mean age at release was 42.0 years (SD = 12.3)	Age at release demonstrated inverse relationship to the probability of sexual and/or violent recidivism.<br>Rates of sexual and violent recidivism declined in older age, with very low rates of recividim in older offender groups.<br>Overall, age at release was negatively correlated with BARS score. Older offenders appeared to be a lower risk group in general, with higher risk older offenders being rare.<br>Age tends to mitigate overall risk, but not all older offenders are low risk.	Aging and risk of reoffending;

**Nilsson et al. (2014) ***** [74]**	Sweden	The aims of this study are: (i) to establish the rate of recidivism among child sexual offenders, (ii) to compare intra- and extra-familial offenders and (iii) to test the predictive ability of common demographic, criminological and clinical characteristics in a prospective, long-term clinical follow-up study of convicted perpetrators of child sexual abuse sentenced to correctional treatment or forensic psychiatric care.	Quantitative Analysis of case file data	Population-based cohort: 196 child sexual abuse perpetrators. Age range: 18-46 (median age = 42) Clinic-referred nationwide study group: 185 cases. Age range 15-75 (median age =40)	Age at first conviction was tested with Receiver Operating Characteristic (ROC) analyses for its ability to predict sexual, non-sexual violent and any criminal recidivism in both main groups. This variable showed a good predictive ability in both groups, especially for violent reconvictions, indicating that the lower the offender's age at first conviction, the higher the risk for recidivism	Aging and risk of reoffending

**Olver, Nicholaichuk, Gu, & Wong (2012) *** [75]**	Canada	An examination of sex offender treatment outcome in a large national cohort of Canadian Federally incarcerated sex offenders followed up an average of 11.7 years post-release.	Quantitative Analysis of case file data and actuarial risk assessment	732 sex offenders 625 were in the treated group; 107 were in the comparison group	Younger offenders had significantly higher sexual recidivism failure rates; No differences between older treated and untreated offenders in sexual recidivism failure rate; Younger offenders reoffended at higher rates irrespective of treatment condition; Significant differences in outcome observed only among younger offenders and treatment completion uniquely predicted reductions in subsequent violence; Differences in outcome and the lack of an interaction explained by the fact that the older offenders were already a lower risk group, both in terms of their actuarial risk as well as older age.	Aging and risk of reoffending; Treatment

**Poortinga, Lemmen & Majeske (2007) ** [76]**	USA	[To] replicate the findings of Firestone et al. (2005) with respect to defendant psychopathology and defendant age, using logistical regression modelling to control for other independent variables that may contribute to the variance in the outcome (dependent) variable of victim age	Quantitative Retrospective, case-control study Examined demographic profiles and patterns of psychiatric morbidity of defendants Review of police reports and medical records	Reviewed charges of approx. 25,000 defendants. Found a total of 294 defendants with a known age of victim	125 participants were aged over 60<br>169 participants aged 18-49<br>Older participants more likely to have younger, weaker victims (more difficult physically restraining or overpowering victims over the age of 12)	Characteristics of Individuals Convicted of Sexual Offenses and the Offense

**Prentky & Lee (2007) ***** [77]**	USA	Examined the hypothesised effect of age-at-release on rates of new sexual charges	Quantitative Analysis of case file data	251 male sex offenders; 136 rapists and 115 child molesters<br>Rapists age at release 21.5 - 80.9 (mean age = 37.24; SD = 11.79)<br>Child molesters age at release 19.95 - 74.9 (mean age = 41.45; SD = 11.82)	Rapists: proportion of sexual re-offence was stable at 28% through to late 30s, reducing to 22% in the 40s, declining in 50s, and reaching 0 (no detected re-offence) in the oldest group aged 60+<br>Child molesters: Pattern quadratic not linear. Youngest group (18-29) had lower sexual re-offence rate, increasing for those released in their 30s, declining slightly for those released in their 40s, and dropping for those released in their 50s and 60s. Decline does not appear until age of 50.	Aging and risk of reoffending
**Proulx et al. (1997) *** [78]**	Canada	To verify whether the recidivism rate in a large sample of sexual aggressors is a function of static or dynamic predictors of the following categories: criminal history, demographic characteristics, and psychometric and phallometric data	Quantitative	382 men with an official record of at least one sexual offence	For violent offences, those who reoffended had a significantly lower mean age than those who did not reoffend violently.<br>Those who reoffended had a significantly lower mean age than those who did not reoffend<br>Reconvicted rapists were younger and had more previous convictions than those who were not reconvicted.	Aging and risk of reoffending

**Rayel (2000) ** [79]**	USA	To determine the clinical and demographic characteristics of elderly sexual offenders	Quantitative Analysis of case file data	7 offenders aged over 55 years<br>Charged with indecent assault and battery or rape	Majority of elderly sex offenders belonged to the 60-69 year old range<br>86% were unmarried at the time of admission; lack of stable sexual partner increased likelihood of acting in a sexually inappropriate way<br>Almost half served in the military<br>The majority had a mood or psychotic disorder. One diagnosed with paraphilia.<br>Violence was present in 28% of offenders	Characteristics of Individuals Convicted of Sexual Offenses and the Offense
**Redondo, Luque, Navarro, & Martinez (2007) *** [80]**	Spain	The aim of the case study […] was to identify and describe the main personal and criminal career features of sexual offenders, as well as the how these are linked to risk of future recidivism.	Quantitative Analysis of case file data	123 individuals convicted of sexual offences<br>49 psychologically treated, 74 not treated.	First sex offence was committed, on average, at age 31.8 years recidivists were significantly younger than non-recidivists on committing their first (convicted) sex offence (25.2 years compared to 34.3 years)	Characteristics of Individuals Convicted of Sexual Offenses and the Offense ;<br>Aging and risk of reoffending

**Rettenberger, Briken, Turner & Eher (2015) ***** [82]**	Austria	To provide a further contribution to the international status of research about sexual offender recidivism by investigating the recidivism rates of sexual offenders in a representative sample released from the Austrian Prison System.	Quantitative; Analysis of national case file data	1,115 registered male sex offenders; 537 child molesters and 532 rapists	Significant negative linear function between age at time of release and violent recidivism for the total sample and for the rapist subgroup Cubic trend within the relationship between age on release and recidivism with two bends in it. The total sample and both subgroups showed decrease in recidivists across the four age bands for violent recidivism, with a stronger aging effect for the rapist subgroup than child molesters. No such effect was found between age and sexual recidivism.	Aging and risk of reoffending

**Rettenberger, Haubner-Maclean, & Eher (2013) ***** [81]**		The purpose of this study was to examine the influence of several age-related variables on the predictive accuracy of the German version of the Static-99 using a population-based sample of prison-released sexual offenders (N = 1,077).	Quantitative; Analysis of Static-99 data and recidivism data	1,077 male sexual offenders;<br>550 child molesters, 501 rapists, and 26 mixed offenders	Age at release did not add incremental validity to the Static-99 for the prediction of sexual recidivism;<br>For the prediction of violent recidivism, the results indicated incremental predictive validity of age at release;<br>Age-corrected Static-99R could not sufficiently capture the effect of age at release on violent recidivism.	Aging and risk of reoffending
**Rodriguez, Boyce & Hodges (2017) ** [83]**	Australia	To investigate executive function, decision-making, and memory in a sample of older adult first-time sex offenders.	Mixed methods Semi-structured interviews;<br>Review of screening, neuropsychological, and executive function measures	100 male subjects aged 50 - 85 (mean = 61.2 years; SD = 7.1)<br>*n* = 32 first time sex offenders (median age = 63)<br>*n*= 36 historical offenders (median age = 62)<br>*n* = 32 non-sex offenders (median age = 55.5)	Depression was the most common psychiatric complaint in first time sex offenders (FTSOs); however, they were not significantly different from the historical long-term sex offender (HSOs) and non-sex offender (NSOs) groups;<br>Older adult FTSOs showed impairment across a range of executive tasks compared to an older NSOs;<br>Older adult sex offenders, overall, demonstrated poorer neuropsychological performance than older adult NSOs did, although there was no difference between older FTSOs and HOs.	Characteristics of Individuals Convicted of Sexual Offenses and the Offense

**Romano & De Luca (1997) ** [84]**	USA	To offer preliminary empirical findings on the nature of any possible relationship between childhood sexual abuse and adult sexual offending behaviour in a sample of male sexual offenders who have had histories of sexual abuse	Quantitative Self-report, questionnaire data	24 male child sexual offenders<br>Age range: 17 - 67 (mean age = 41)	Trend between onset of sexual offending and offenders' own sexual abuse.<br>Most sexual offenders (44%) indicated that their first childhood sexual abuse experience was with an individual who was older than 21.<br>Majority of sexual offenders (50%) were reportedly older than 21 when they committed their first CSO	Characteristics of Individuals Convicted of Sexual Offenses and the Offense

**Swinburne Romine, Miner, Poulin, Dwyer & Berg (2012) **** [85]**	USA	To explore the factors that predict reoffending in this sample of community-based sexual offenders, which includes substantial numbers of both hands on and hands off offenders	Quantitative Analysis of case file data	744 participants<br>718 male, 14 female, rest unknown	In the group of 55 or older (n = 73), 4% reoffended by sexual contact, 3% noncontact sexual<br>rate of reoffending for contact sexual offenses and for any offenses was stable from below 25 years of age to 45 years of age.<br>A significant decrease in reoffence of any type was seen for those offenders who were 45 or older at the beginning of their time at risk<br>No increase in re-offense was seen for those participants below the age of 25 at the beginning of their time at risk. In fact, we found a consistent level of reoffending from below 25 to 45 years of age, where there was a dramatic drop.	Aging and risk of reoffending

**Ryan, Wilson, Kilgour & Reynolds (2014) ***** [86]**	New Zealand	To identify whether it was possible to modify the Public Health Assessment (PHA) referral guidelines so that fewer false- positive PHA referrals (i.e., referrals made for a PHA in which the recommendation made is not to recommend an ESO) are made with no change (or an improvement) to the sensitivity/accuracy of the proportion of cases identified as potentially eligible for an extended supervision order.	Quantitative Analysis of ARSRS measure items, PHA reports and conviction history	182 offenders assessed for an extended supervision order between 2004 and 2008	Older men more likely to be recommended for an extended supervision order due to having a history of repeated sexual deviance; The older the offender, the more time he has had to incur further sexual and general offences, which can elevate his score on dynamic risk measures; Older men with higher ASRS scores tend to demonstrate a greater number of dynamic risk factors than their younger counterparts.	Aging and risk of reoffending
**Seto & Eke (2015) ***** [87]**	Canada	To identify risk factors for recidivism among child pornography offenders using a richer dataset than the registry follow-up reported by Eke et al. (2011).	Quantitative Analysis of Police case file data	266 adult male child pornography offenders in the community	CP offenders: risk factors are consistent with established models of contact sexual offending that emphasise antisocial propensities (e.g. younger age) In the fixed 5-year follow up, seven significant predictors of any sexual recidivism were identified, including offender age at time of index investigation	Aging and risk of reoffending

**Sjöstedt & Långström (2001) ***** [88]**	Sweden	To cross-validate the RRASOR and the Static-99 in a different sociocultural and legal context using a large nationwide cohort of all sex offenders released from prison in Sweden during a 5-year period	Quantitative RRASOR and Static-99 data Outcome measure was a registered sexual reconviction, or any violent reconviction.	1,400 men convicted of a sexual offence, served a prison sentence and released between 1993 - 1997 Age range: 18-77 (mean = 40.23, SD = 11.99)	Young age not related to sexual recidivism risk but associated to an elevated risk of any violent recidivism.	Aging and risk of reoffending
**Skelton & Vess (2008) *** [89]**	New Zealand	Examined sexual re-offending as a function of age and actuarial risk in a large sample of sexual offenders released from prison between 1990 and 2004	Quantitative ASRS data	5,880 sex offenders Age range: 15 - 85 (mean = 40.0 years; SD = 14) 31% offended against children under age of 16 54% offended against adults 15% offended against both adults and children	Younger age groups re-offending at a higher rate in comparison to offenders over age 50. Older age-at-release groups began sexually offending at a progressively older age. Except the youngest and oldest age groups, those who sexually re-offend had a significantly younger age at first sexual offence compared to those who did not sexually re-offended While sexual re-offences became less frequent with age in general, those with the highest static risk accounted for a disproportionate number of these re-offences until after age 60.	Aging and risk of reoffending

**Smallbone & Wortley (2004) *** [91]**	Australia	To examine onset and persistence of sexual and nonsexual offending among those offenders in the original sample who (a) admitted to at least one sexual offense against a child, and (b) provided all required self-report data (n = 207).	Quantitative Analysis of case file data/self-report questionnaire	207 adult males convicted of sexual offenses against children.<br>Mean self-reported age of first sexual contact with child = 32.3 years (age range = 10 - 63)<br>Mean age at first conviction (of any offense) = 30.5 years (range = 12 - 66)<br>Mean age at first conviction for a sexual offense = 37.3 years (range = 15 - 76)	Moderate to strong positive correlations were obtained between the three onset variables (self-reported age at first sexual contact with a child, age at first conviction for a sexual offences, and age at first conviction for any offence)<br>Small negative correlations between age at first sexual contact with a child and number of sexual offence victims, number of sexual offence convictions, and number of nonsexual offence convictions.<br>A moderate negative correlation was found between age at first conviction for any offence and the number of nonsexual convictions, and a small negative correlation was found between age at first sexual offence conviction and the number of nonsexual offence convictions.<br>The mean age at first sexual contact with a child was 32.3 years (median = 31). Onset ages were normally distributed across a wide range (10-63).<br>Extrafamilial offenders reported an earlier onset than intrafamilial offenders.<br>Intrafamilial were significantly older at when first convicted of a CSO than both extrafamilial and mixed-type offenders<br>Offenders were significantly older at the time of their first sexual contact with a child than they were at the time of their first conviction of any offence, and significantly older when first convicted of a SO than time at first conviction for any offence.	Characteristics of Individuals Convicted of Sexual Offenses and the Offense

**Smallbone, Marshall & Wortley (2008) [90]**	Australia	N/A	N/A	N/A	Two distinct populations based on age of onset of sexual offending: (1) individuals who first sexually offended during adolescence but stopped offending into adulthood, and (2) most adult offenders who start to offend during adulthood had a peak risk period of late thirties.	Characteristics of Individuals Convicted of Sexual Offenses and the Offense
**Stephens, Cantor, Goodwill & Seto (2017) ***** [92]**	Canada	The study examined the relationship between sexual interest in prepubescent children (pedophilia) or pubescent children (hebephilia) and sexual recidivism	Quantitative Assessment and case file data	656 males who committed a sexual offense<br>Mean age = 41.1 years (SD = 13.0).<br>Mean age at first sexual offense was 30.3 years (SD = 13.0).<br>Mean age at most recent sexual offense = 37.4 years (SD = 12.7)	Younger age at time of assessment was associated with sexual contact and violent recidivism, but not noncontact sexual recidivism.	Aging and risk of reoffending

**Sullivan & Beech (2004) ***** [93]**	UK	[To] explore the demographic information available on a group of professional perpetrators and compare this with a population of child sexual abusers containing both intra and extra-familial perpetrators to establish whether the professional perpetrators appear to have different characteristics to the other abusers	Mixed methods Questionnaire using the National Organisation for the Treatment of Abuse (NOTA) database questionnaire and semi structured interviews	305 participants	If this study group are representative of all professional perpetrators, then they are likely to be older and more intelligent than other sex offenders. Oldest participants is the faith community leaders with a mean age of 53.15 (SD 10.87). The group of teachers had a mean age of 49.10 (SD 14.33) and the youngest group was the childcare workers with a mean age of 35.25 (SD 6.18).	Characteristics of Individuals Convicted of Sexual Offenses and the Offense

**Thornton (2006) ***** [94]**	United Kingdom	[To examine] the relationship between age and sexual recidivism over a broad age range	Quantitative National case file data	752 imprisoned male sex offenders Followed-up for 10 years	Age positively correlated with number of prior sexual sentencing occasions, and negatively with a history of nonsexual offending; those released at a younger age tended to be general offenders, whereas those released at a later age were more likely to be sexual specialists.<br>The odds of sexual reconviction declined by 0.02 with each year of increasing age.<br>Confirms inverse relationship between age of release and sexual reconviction.<br>For those with one prior sexual sentencing, the effect of age was characterized as gradual linear decline in the odds of sexual recidivism. For those with no prior sexual sentencing occasions, age on release and sexual recidivism were unrelated.<br>For those with two or more prior sex offences, there was a significant cubic trend; the graph changed slope at two points. High sexual recidivism rate between 18 and 25, a reduction (from 80% to 50%) for offenders released after that age, with no decline in sexual recidivism until the ago of 60 when the rate falls by approx. 40%	Offender and offense characteristics; Aging and risk of reoffending

**Tsagaris, Bach & Cimino (2017) ** [95]**	USA	This study focuses on the crimes of sexual exploitation of children facilitated through the Internet.	Quantitative Analysis of case file data	24 offenders convicted of child sex offences Age range: 22 - 68 (mean age = 39.8)	Older offenders tended to have higher educational levels and more experienced employment history; Older individuals had more time in which to generate a substantive employment and educational history.	Characteristics of Individuals Convicted of Sexual Offenses and the Offense
**van Gijn-Grosvenor & Lamb (2016) ***** [96]**	USA	This study qualitatively and quantitatively described the ways 101 convicted offenders behaved when grooming boys or girls online for offline sexual encounters	Quantitative Experiment	101 male offenders convicted of grooming children online for sexual purposes Age range = 19 - 69 (mean age = 38.11; SD = 12.27)	Offenders grooming girls (median = 29) were significantly younger than offenders approaching boys (median = 42)	Characteristics of Individuals Convicted of Sexual Offenses and the Offense
**Wakeling, Freemantle, Beech, & Elliott (2011)***** [97]**	UK	To identify the best predictors of sexual and violent recidivism in a group of treated sexual offenders.	Quantitative Risk assessment data (RM 2000)	3,773 sexual offenders	Age at release, and younger age, was a predictor of recidivism.	Aging and risk of reoffending

**Willis & Ward (2011) **** [98]**	New Zealand	To explore the practical utility of the GLM with a sample of released child molesters, and investigate the relationship between primary goods attainment and overall re-entry conditions (in terms of accommodation, social support and employment)	Quantitative Semi-structured interviews	16 child molesters interviewed at three different timepoints after release (1,3 and 6 months)	Significant positive correlations between offender age and good lives ratings at one month and three months post-release; older participants tended to have higher good lives ratings. Significant negative correlation between offender age and Stable-2007 scores, meaning that older participants tended to have lower assessed levels of dynamic risk factors	Aging and risk of reoffending

**Woodworth et al. (2013)** ***** **[99]**	Canada	To answer a number of important questions pertaining to high-risk sexual offenders. Specifically, we sought to answer the following: (1)What is the prevalence of deviant sexual fantasy among high-risk offenders?; (2) Is there a relationship between offenders' fantasy themes and the offences they perpetrate?; (3) Is there a relationship between level of psychopathy and the sexual fantasies that offenders report?; (4) What is the prevalence of sexual paraphilia among high-risk offenders?; (5) Do the number and type of paraphilias differ across offender typology?; and (6) Is there a relationship between level of psychopathy and the paraphilia(s) that offenders report?	Quantitative Analysis of case file data	139 high-risk male sex offenders<br>Age range: 19 to 77 years (Mean = 43.88, SD = 11.24)	Offender age at first adult sexual offence ranged from 18 to 55 years<br>The age of offenders' first nonsexual offence ranged from 11 to 53 years, with 18 offenders reporting no history of nonsexual offences;<br>Mixed offenders (engaged different types of sexual offences or who were indiscriminate for victim age) had first conviction at a much younger age than offenders who victimized children exclusively; although child molesters began offending later (or were first convicted later in life) than other offender types, they are more persistent in their offenses as evidenced by their greater number of sexual convictions and victims	Characteristics of Individuals Convicted of Sexual Offenses and the Offense

**Wortley & Smallbone (2014) ***** [100]**	Australia	To apply a criminal careers model to child sexual abusers	Quantitative Review of official data and self-report data on personal and offending histories	362 convicted offenders, 213 provided self-report data	Limited/specialized offenders: relatively older at the time of first sexual contact with child; Waited until early 30s before first offense; abuse of power/trust; already have access to victim.<br>Persistent/versatile offenders: Involvement in criminal justice system from an early age and earlier age for first sexual contact with a child.<br>Persistent/specialist: earlier sexual contact with child; involvement in criminal justice system begins at later age (earlier than limited specialists).	Characteristics of Individuals Convicted of Sexual Offenses and the Offense

Table 1: Summary information of included studies (n = 100)

***Note**: M = Mean; SD = Standard Deviation; n = sample size; Static-99R (Helmus, Thornton, Hanson & Babchishin, 2012); Static 2002R (Hanson & Thornton, 2003); BARR-2002R (Babchishin, Hanson & Blais, 2013); PCL-R (The Hare Psychopathy Checklist-Revised; Hare,1991); MATS-1 (Multisample Age-Stratified Table of Sexual Recidivism; Wollert, Cramer, Waggoner, Skelton & Vess, 2010); Static-99 (Hanson & Thornton, 2000); RM 2000 (Risk Matrix 2000; Thornton et al., 2003); RRASOR (Rapid Risk Assessment for Sexual Offense Recidivism; Hanson, 1997); Static-2002 (Hanson & Thornton, 2002); BARS (Brief Actuarial Scale; Olver, Nicholaichuk, Gu & Wong, 2013).*

*The number of * after the Author denotes the ‘Mixed Methods Appraisal Tool’ (MMAT; Hong et al., 2018) rating that was given to each study;1 and 2 stars are referred to as low quality studies, and 4 and 5 stars are higher quality studies.*

The number stated in the [X] after the MMAT ratings refers to the reference table (Table 1) provided in the main Systematic Literature review. The table provides details pertaining to the studies that have been included in each theme
